# Learning Multisensory Integration and Coordinate Transformation via Density Estimation

**DOI:** 10.1371/journal.pcbi.1003035

**Published:** 2013-04-18

**Authors:** Joseph G. Makin, Matthew R. Fellows, Philip N. Sabes

**Affiliations:** Department of Physiology and the Center for Integrative Neuroscience, University of California San Francisco, San Francisco, California, United States of America; Queen's University, Canada

## Abstract

Sensory processing in the brain includes three key operations: multisensory integration—the task of combining cues into a single estimate of a common underlying stimulus; coordinate transformations—the change of reference frame for a stimulus (e.g., retinotopic to body-centered) effected through knowledge about an intervening variable (e.g., gaze position); and the incorporation of prior information. Statistically optimal sensory processing requires that each of these operations maintains the correct posterior distribution over the stimulus. Elements of this optimality have been demonstrated in many behavioral contexts in humans and other animals, suggesting that the neural computations are indeed optimal. That the relationships between sensory modalities are complex and plastic further suggests that these computations are learned—but how? We provide a principled answer, by treating the acquisition of these mappings as a case of density estimation, a well-studied problem in machine learning and statistics, in which the distribution of observed data is modeled in terms of a set of fixed parameters and a set of latent variables. In our case, the observed data are unisensory-population activities, the fixed parameters are synaptic connections, and the latent variables are multisensory-population activities. In particular, we train a restricted Boltzmann machine with the biologically plausible contrastive-divergence rule to learn a range of neural computations not previously demonstrated under a single approach: optimal integration; encoding of priors; hierarchical integration of cues; learning when not to integrate; and coordinate transformation. The model makes testable predictions about the nature of multisensory representations.

## Author Summary

Over the first few years of their lives, humans (and other animals) appear to learn how to combine signals from multiple sense modalities: when to “integrate” them into a single percept, as with visual and proprioceptive information about one's body; when *not* to integrate them (e.g., when looking somewhere else); how they vary over longer time scales (e.g., where in physical space my hand tends to be); as well as more complicated manipulations, like subtracting gaze angle from the visually-perceived position of an object to compute the position of that object with respect to the head—i.e., “coordinate transformation.” Learning which sensory signals to integrate, or which to manipulate in other ways, does not appear to require an additional supervisory signal; we learn to do so, rather, based on structure in the sensory signals themselves. We present a biologically plausible artificial neural network that learns all of the above in just this way, but by training it for a much more general statistical task: “density estimation”—essentially, learning to be able to reproduce the data on which it was trained. This also links coordinate transformation and multisensory integration to other cortical operations, especially in early sensory areas, that have have been modeled as density estimators.

## Introduction

The brain often receives information about the same feature of the same object from multiple sources; e.g., in a visually guided reach, both vision and proprioception provide information about hand location. Were both signals infinitely precise, one could simply be ignored; but fidelity is limited by irrelevant inputs, intrinsic neural noise, and the spatial precisions of the transducers, so there are better and worse ways to use them. The best will not throw away any information—in Bayesian terms, the posterior probability over the stimulus given the activities of the integrating neurons will match the corresponding posterior given the input signals. Encoding in the integrating neurons the entire posterior for each stimulus, and not merely the best point estimate, is crucial because this distribution contains information about the confidence of the estimate, which is required for optimal computation with the stimulus estimate [1], [2]. A sensible code will also “compress” the information—for example, by representing it in fewer neurons—otherwise the brain could simply propagate forward independent copies of each sensory signal.

Psychophysical evidence suggests that animals—and therefore their brains—are indeed integrating multisensory inputs in such an “optimal” manner. Human subjects appear to choose actions based on the peak of the optimal posterior over the stimulus, given a variety of multisensory inputs [1], [3]–[7]. Prism and virtual-feedback adapation experiments [8]–[12] have demonstrated the plasticity of these multisensory mappings, and it is not likely limited to recalibration: Deprivation studies [13]; afferent re-routing experiments [14], [15]; the ability to learn novel, cross-modal mappings; and genetic-information constraints together suggest that integration is learned, with the organization of association cortices driven by sensory data.

A plausible neural model of multisensory integration, then, must learn without supervision how to combine optimally signals from two or more input populations as well as *a priori* information, encoding both the most likely estimate and certainty about it—even when the relationship between the signal spaces is nonlinear (like retinotopic and proprioceptive-encoded hand location), and when their relationship is mediated by another variable (like gaze angle). Existing computational models of multisensory integration or cross-modal transformation neglect one or more of these desiderata (see Discussion).

Here we show that the task of integration can be reformulated as *latent-variable density estimation*, a problem from statistics that can be implemented by a neural network, and the foregoing requirements thereby satisfied. The goal is to learn a data distribution (here, the activities of populations of visual and somatosensory neurons while they report hand location in their respective spaces) in terms of a set of parameters (synaptic strengths) and a set of unobserved variables (downstream, integrating neurons). In particular, we model the cortical association area with a restricted Boltzmann machine (RBM), an undirected generative model trained with a fast, effective Hebbian learning rule, contrastive divergence [16], [17]. By making the machine a good model of the distribution of the training data, learning obliges the downstream units to represent their common underlying causes—here, hand location. The same formulation turns out to be equally suited to coordinate transformation as well.

## Results

**Figure 1:**
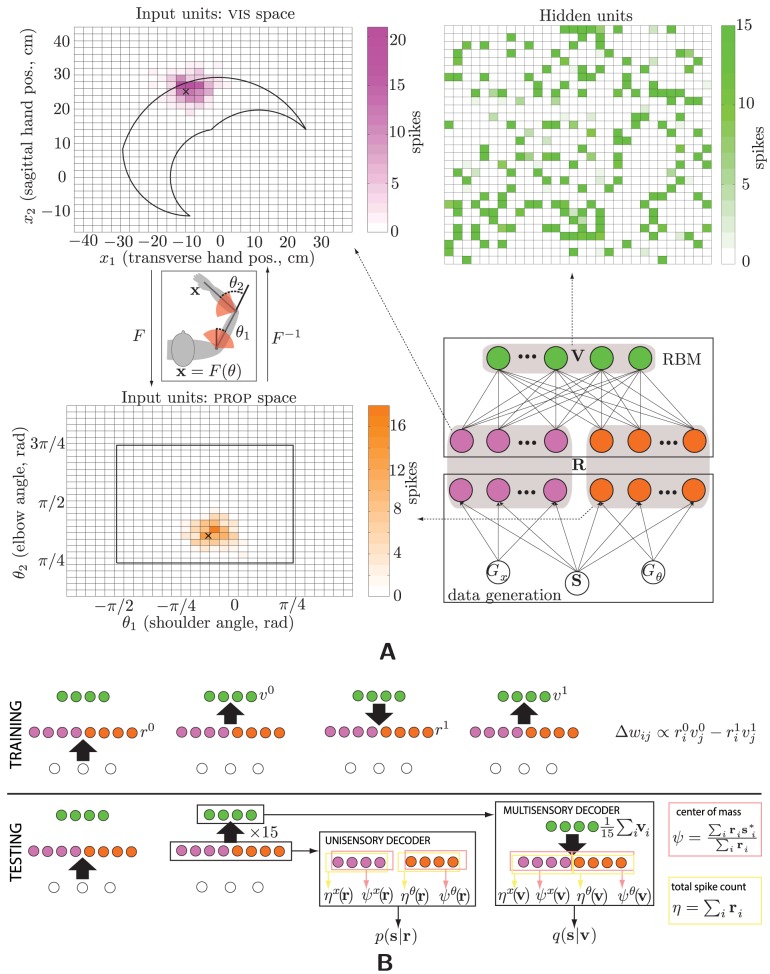
Multisensory integration: data and model. (A) The model and example data. World-driven data are generated according to the directed graphical model boxed in the lower right: On each trial, a hand location 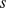 and the population gains 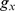 and 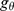 for the two sensory modalities are drawn from their respective prior distributions. Given these, a spike count is drawn for each neuron (magenta and orange colored circles) from a Poisson distribution (see Eq. 2), yielding (e.g.) the set of firing rates shown by the heat maps at left. The center of mass of each population is marked with an x. The visual (magenta) and proprioceptive (orange) neural populations each encode the location of the hand, but in different spaces: 2D Cartesian space and joint space, respectively, drawn in outline in the heat maps. Since the neurons' preferred stimuli uniformly tile their respective spaces (indicated by the grids), but the forward kinematics relating these variables is nonlinear (inset; joint limits are indicated with red shading, joint origins with black lines), hand position is encoded differently by the two populations. These population codes also constitute the input layer, **R**, of the RBM (lower right). Its hidden units, **V**, are Bernoulli conditioned on their inputs, corresponding to the presence or absence of a spike. The green heat map in the upper right depicts the mean of 15 samples from the hidden layer of a trained network for the example inputs shown at left. (B) Testing and training. In the first step of training (first panel), the external world elicits a vector of Poisson spikes from the input layer, driving recurrent activity in the neural network—up, down, and back up (second through fourth panels). The weights are then adapted according to the one-step contrastive-divergence rule. Testing also begins with a world-driven vector of Poisson spikes from the input populations, which drives 15 samples of hidden-layer activity (second panel). We then decode the input and hidden layers, yielding their respective posterior distributions.

A network that has learned to perform the integration task will transmit to downstream neurons (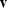), on each trial, all the information in its inputs (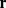) about the stimulus (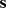). In our case, that network is the RBM, the stimulus is the location of the hand, and the inputs are two neural populations (visual and proprioceptive) encoding hand location in different spaces (Fig. 1A; see also **Methods**). Equivalently, integration requires that the posterior distribution over the stimulus given the activities of the downstream (“hidden” or “multisensory”) units, 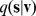, match the posterior over the stimulus given the two inputs, 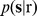. Henceforth, we call the latter of these distributions the *optimal posterior*, since it serves as the benchmark for performance. Having arranged, by our choice of input-population encoding, for the optimal posterior to be Gaussian (see **Methods**), its statistics consist only of a mean and a covariance. Thus to show that the network successfully integrates its inputs, we need show only that these two cumulants can be recovered from the multisensory neurons—intuitively, that they have learned to encode the optimal stimulus location and confidence in that location, respectively. We emphasize that throwing away covariance (or other statistical information) would render subsequent computations suboptimal: for example, if the integrated estimate is itself to be integrated downstream with another modality, it must be weighted by its own precision, i.e. inverse covariance (see **Text S1** and **Hierarchical networks** below).

### Multisensory integration in the RBM

We begin by examining the ability of our model to perform optimal multisensory integration, in the sense just described. We use our “standard” network, with a visible layer of 1,800 Poisson units, comprising two 30×30 input populations, and a hidden layer of half that number of Bernoulli units. We trained and tested this network on separate datasets, with stimuli chosen uniformly in the 2D space of joint angles (see **Methods** and Fig. 1B).

#### Decoding the posterior mean

**Figure 2:**
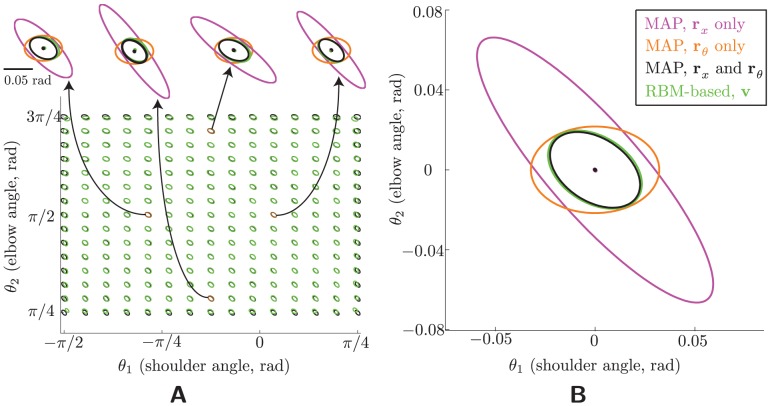
Recovery of the posterior mean. The four ellipses in each plot correspond to the covariances of four different estimates of the stimulus: the MAP estimate of the stimulus using only the visual input population (magenta), the MAP estimate using the proprioceptive input population (orange), the MAP estimate using both populations (i.e., the true posterior mean, which is the optimal estimate; black), and the estimate based on decoding the hidden layer (“RBM-based estimate”; green). (The color conventions are the same throughout the paper.) Each ellipse bounds the 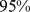 confidence interval and is centered at its mean. All results are shown in the space of joint angles in units of radians. (A) Conditional errors. The middle plot shows the conditional errors for a grid of stimulus locations (each centered at the true stimulus); four examples are enlarged for clarity. Note that nontrivial biases arise only at the edges of the workspace. (B) Marginal error statistics. The RBM-based error (green) is unbiased and its covariance closely matches the optimal covariance (black).

We first show that the hidden layer successfully encodes the optimal-posterior mean. For a fixed stimulus location, 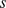, we compare the distribution of the stimulus decoded from 15 samples of the hidden units, 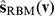 (“RBM-based estimate”, see **Methods**), with the distribution of the optimal-posterior mean, 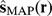. (The latter estimate also has a distribution across trials, even for a fixed stimulus, because the input encodings are noisy.) We compare the distributions of these two estimates, rather than simply examining the distribution of their difference, because the resulting figures (Fig. 2A) then resemble those typically presented in psychophysical studies, where behavior plays the role of the estimate—and indeed, has been found to correspond to the optimal-posterior mean [1].

Fig. 2A shows the mean and covariance of the conditional estimator distributions, 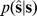, for various stimulus locations 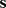, and for four separate estimates of the posterior mean: the MAP estimate using the visual-population activities (magenta), the MAP estimate using the proprioceptive-population activities (orange), the MAP estimate using both input populations (the “optimal” posterior mean, black), and the estimate using the hidden-layer activities (“RBM-based integrated estimate,” green). Each ellipse depicts the 95% confidence interval of the distribution's covariance, centered at its mean, as in all subsequent figures. Clearly, the RBM-based estimate matches the MAP estimate over nearly all of the workspace. Visible errors occur only at the edges of joint space, probably a result of the “edge effects,” i.e., the proximity of extreme joint angles to regions of space not covered by the (perforce finite) grid of neurons.

We can quantify the contribution of these imperfections to the total optimality of the model. Since the MAP estimate is the unique minimizer of the average (over all stimuli) mean square error, the *marginal error distribution*, 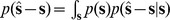, summarizes all the conditional estimator distributions. These marginal error statistics (Fig. 2B stdmargstats) show that the overall performance of the network is very nearly optimal.

#### Decoding the posterior covariance

We next show that the hidden layer also encodes the optimal-posterior covariance. The posterior covariance represents the uncertainty on a single trial about the true stimulus location, given the specific spike counts on this trial. Since *on a single trial*, only one point from the posterior distribution (presumably the mean) manifests itself in a behavior—e.g., a reach—, that trial's posterior covariance cannot be read off the behavior as the posterior mean can. Nevertheless, the posterior covariance has important behavioral consequences *across* trials: it determines the relative weighting of each input during optimal integration (see Eq. 3b in **Methods**). This is clearly a requirement for the input populations, vis and prop; but if, for example, the multisensory (hidden-unit) estimate, 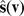, is itself to be integrated with yet another sensory population at a further stage of processing, optimality of that integration requires knowledge of the posterior covariance, in order to weight 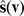 properly. We show in **Hierarchical networks** below that the model can learn just such an architecture, demonstrating that posterior covariance information is indeed encoded in the hidden units; but here we exhibit the result more directly.

The posterior precision (inverse covariance) on each trial is a 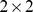 symmetric matrix and therefore ostensibly has three degrees of freedom. However, as shown below in Eq. 3a (**Methods**), the encoding scheme constrains it to a lower-dimensional manifold: the only quantities that change from trial to trial are the “total spike counts,” 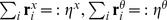, and the location where the Jacobian of the forward kinematics is evaluated. The latter is given by the posterior mean, which we have just shown can be reconstructed nearly optimally. Therefore, reconstruction of the posterior precision requires the additional recovery only of the total spike counts of the respective input populations.

**Figure 3:**
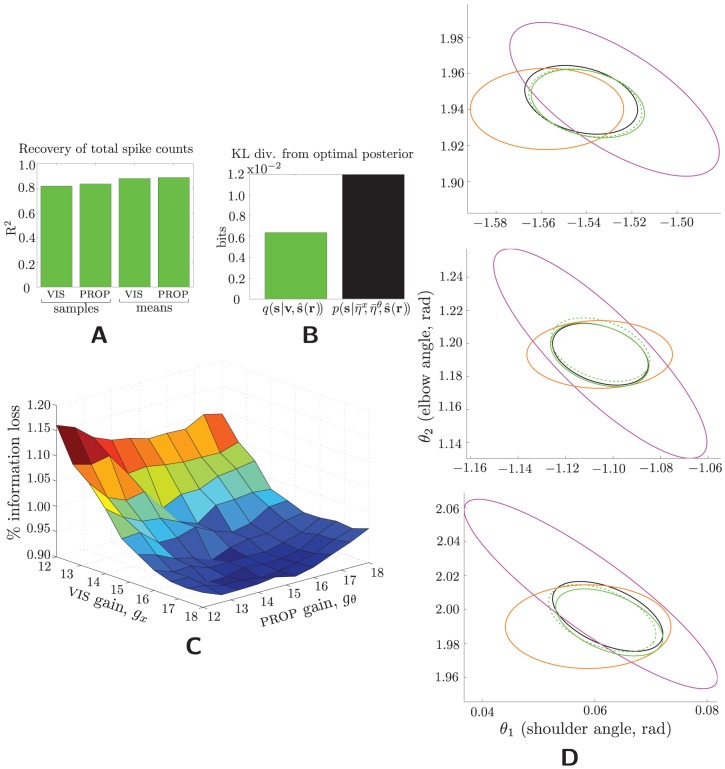
Recovery of the posterior distribution. (A) Reconstruction of the input total spike counts, 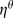 and 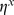, for vis and prop, resp., from 15 samples of the hidden units (“samples”), and from infinite samples of the hidden units (“means”). Decoding these, along with the posterior mean (demonstrated in Fig. 2), is sufficient to recover to posterior covariance. (B) Average (across all trials) KL divergences for two distributions from the optimal posterior, 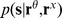: (black) the posterior over **s** given the mean (across trials) total spike counts (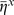 and 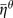) and the optimal posterior mean, 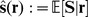; and (green) the sample-based model-posterior, given also the optimal posterior mean. The mean-based model posterior, not shown, is visually indistinguishable. This measures purely the divergence resulting from failure to pass covariance information on to the hidden units. That the RBM-based posterior is so much smaller demonstrates that the model is not merely passing on mean spike-count information, but their trial-by-trial fluctuations. (C) Percent of total information lost from input to hidden units (measured by normalized KL divergence between the optimal and RBM-based posteriors; see **Text S1**), as a function of gains. Information loss is less than about 1.2% for all gains. (D) Posterior distributions (means and covariances) from three randomly selected trials. Color scheme is as throughout; dashed green shows the posterior computed from hidden-unit means (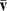), as opposed to samples (**v**, solid green).

Fig. 3A shows the coefficients of determination (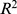) for two different estimators of the total spike counts, one using 15 samples from the hidden-layer units (as for the posterior mean above), and the other using hidden-layer means (i.e., infinite samples; see **Methods**). In all cases, 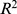 values are greater than 0.82, with the infinite-sample decoder approaching 0.9.

How do these values translate into the quantity we really care about, the posterior covariance, and by implication the posterior distribution itself? To quantify this, we employ the standard measure of similarity for distributions, the KL divergence. Since the true posterior is Gaussian, and since the RBM encodes the (nearly) correct mean and variance of 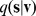, it too must be (nearly) Gaussian. (Given a specified mean and finite variance, the maximum-entropy distribution is normal. Thus if 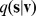 and 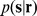 have identical mean and variance, but the latter is Gaussian while the former is not, then the former has lower entropy—which is impossible, since information about 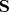 cannot be *gained* in the transition from **R** to **V**.) The KL divergence between two Gaussian distributions has a very simple form, and in fact we make it simpler still by examining only that portion contributed by the covariances—i.e., ignoring mean differences, since we have just examined them in the previous section: 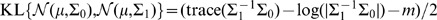, where 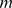 is the number of dimensions. The first bar of Fig. 3B show this divergence from the optimal posterior to the RBM-based posterior (again based on 15 samples).

What constitutes a proper point of comparison? Consider a fixed computation of the covariance which uses Eq. 3a but using the *average* (across all trials) total spike counts, 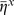 and 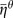, rather than their trial-by-trial counterparts. If the model had learned the prior distribution over the total spike counts, but was not actually encoding any trial-by-trial information, it could do no better than this fixed computation. The KL divergence of the optimal posterior from this fixed computation is shown in the second bar of Fig. 3B. The model is clearly far superior, demonstrating that it is indeed transmitting trial-by-trial covariance information.

Finally, we directly demonstrate the fidelity of the entire model posterior, 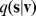, to the entire optimal posterior, 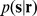, as a function of the population gains, by calculating the fractional information lost in terms of the normalized KL divergence:
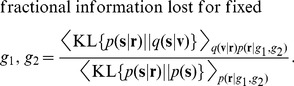
(1)


This quantity is 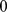 in the best case, when 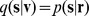, and 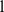 in the worst, when 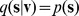. (See also **Text S1** for a more extended discussion.) Fig. 3C shows that slightly more information is lost at low visual gains, but that in fact the slope is very shallow, since all information losses are between the small amounts of 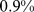 and 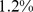. To visualize this small discrepancy, Fig. 3D provides a qualitative comparison of the single-population, dual-population (optimal), and RBM-based posterior distributions, for three random trials. (These are not to be confused with the distribution of the posterior mean, as in Fig. 2A, which is measured *across* trials.) The match between model and optimal posterior is evident for both covariance (size and shape of the ellipse) and mean (its location).

#### Effects of hidden-layer size and hidden-layer noise

Figs. 2 and 3 have shown model performance to be “nearly” optimal, in that both the posterior mean and the posterior covariance are encoded in the hidden layer. The small deviations from optimality can result from two distinct causes: (1) the network having failed to learn the ideal information-preserving transformation, and (2) the noise in the hidden layer having corrupted that transformation. In order to gauge the relative contribution of the two, we re-tested the model under a range of different capacities and noise levels by varying the number of hidden units and the number of samples taken at the hidden layer, respectively. Note that since the hidden units are Bernoulli, increasing the number of samples is akin to increasing the time window over which mean rates of activity are computed. Our assay is the error in the RBM-based estimate of the posterior mean; and since we observe that only the size, rather than the shape or position, of the error-covariance ellipse is greatly distorted as a function of decreasing samples, for simplicity we plot only the determinant of the error-covariance matrix.

**Figure 4:**
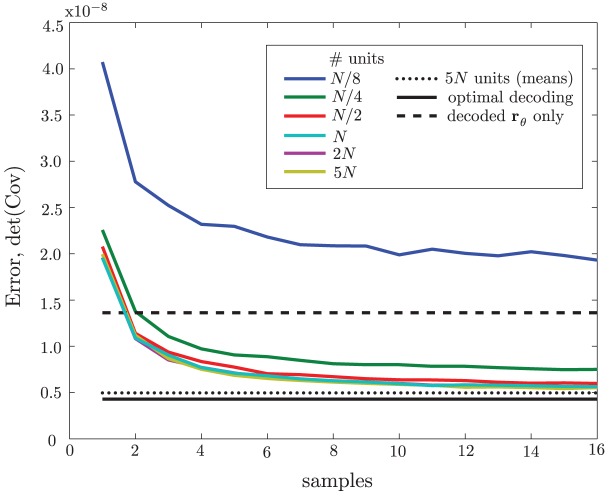
Dependence of error covariance on numbers of samples and hidden units. Networks with different numbers of hidden units (see legend; 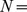 number of units in a single input population) were trained on the input data, and then decoded for the posterior mean in the usual way but using different numbers of samples from the hidden layer (abscissa) before averaging. The determinants of the resulting error covariances are plotted here with colored lines. Dashed line, MAP error covariance using only proprioceptive input; solid line, optimal error covariance; dotted line, error covariance from the 5N network when using means in the hidden layer—i.e., infinite samples—the asymptote of the colored lines.

Fig. 4 shows that, as expected, the error measure decreases both with more hidden units and more samples. However, a comparison of the different curves shows that the error asymptotes at 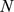 hidden units (cyan line), which is the number of units in one input population—increasing the hidden layer beyond that has no effect on performance. Performance also asymptotes at around ten samples per unit. At asymptote, the errors are close to optimal (solid black line), and much better than the single-input (PROP) error (dashed black line). (The VIS determinant is much larger and therefore omitted.).

Fig. 4 also shows the error for a network with 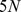 hidden units and the use of means (equivalent to taking infinite samples) in the hidden layer (dotted black line). This error lies about halfway between the optimal and asymptotic RBM-based errors, showing that about half that network's suboptimality is due to noise, and half due to network architecture and the learning algorithm; but in any case the network performance is quite close to optimal.

### Simulating psychophysical studies of optimal integration

We now relate our model to some familiar results from psychophysical investigations of multisensory integration. In the foregoing simulations, the input populations were driven by the same stimulus. The most common experimental probe of integration, however, is to examine the effects of a small, fixed discrepancy between two modalities—with, e.g., prism goggles or virtual feedback [1], [3], [4], [18]–[20]. Integrated estimates tend to fall between the means of the discrepant inputs, revealing the relative weighting of the two modalities. The mean location of the integrated estimate therefore allows experimenters to assess integration without having to obtain reliable estimates of the error covariance. Notice this point will not necessarily lie along the straight line connecting the input means, since the sensory covariances need not be aligned [1].

To replicate these experiments, the trained network from Fig. 2 was tested on sets of “shifted” data in which joint angles had been displaced from their corresponding visual locations by a fixed quantity, the “input discrepancy,” before being encoded in the prop population. To determine how large to make this discrepancy, we returned to the original, unshifted data. Although the *average* discrepancy between the two inputs in this data set is zero (as seen in the locations of the magenta and orange ellipses in Fig. 2), the noisy encoding renders the discrepancy on *single trials* non-zero, with the probability of finding such a discrepancy determined by the sum of the input covariances, 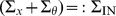. This quantity providing, then, a natural measure of discrepancy, each set of shifted data was created with an input discrepancy of 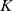 standard deviations of 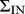, with 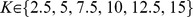. Note that large 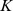 enables a further investigation—into the *generalization* of the trained network: The extent to which the RBM's optimality is maintained as the input discrepancy grows indicates, qualitatively, the generalization powers of the machine on these data.

**Figure 5:**
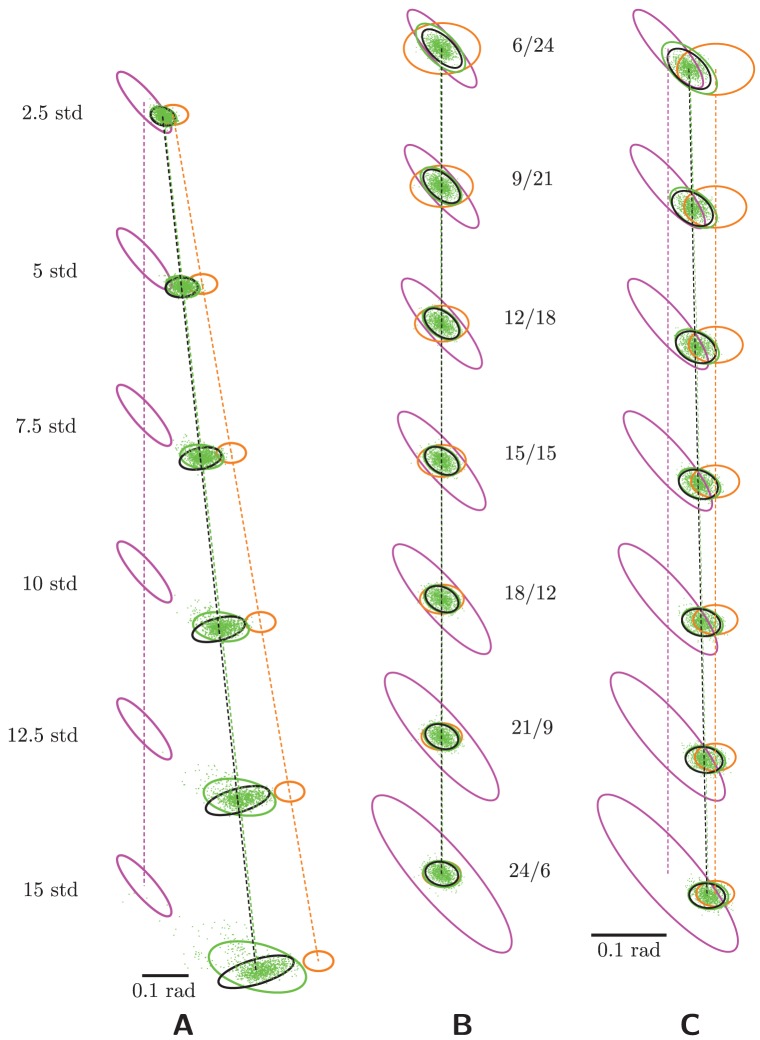
Model generalization across input discrepancies and input gains. After training, the model was tested on data that differ from its training distribution. (A) Discrepant-input data: prop input (orange) is shifted by progressively greater increments of the input covariance (see text), leading to suboptimal integration, as expected, and structured error distributions. The hidden-layer error mean, like the optimal error mean, shifts rightward with the prop “bias.” (B) Gain-modulated data: The training data had gains between 12 and 18. Testing on gains (ratios listed between panels (B) and (C)) outside this regime yields suboptimal error covariances but essentially zero biases. (C) Gain-modulated, input-discrepant data: As the relative reliability of PROP is increased, the optimal estimate shifts toward PROP and away from VIS. The green and black dotted lines, nearly identical, trace this movement for the machine-based and optimal estimates, resp. For larger discrepancies (not shown), this optimal behavior breaks down, the green and black lines diverging.

Fig. 5A shows the error statistics for these testing datasets for several discrepancy magnitudes along a single direction (discrepancies along other directions, not shown, were qualitatively similar). Psychophysicists examine conditional errors, but again for generality we have averaged across stimulus locations to produce marginal errors. The RBM-based estimator (green) becomes noticeably suboptimal by 7.5 standard deviations. Furthermore, the distribution of errors becomes distinctly non-normal for large input discrepancies, spreading instead over the arc connecting the centers of the input error distributions. This arc corresponds to the location of the optimal estimate for varying relative sizes of the input error covariances [1]. Whether such a pattern of errors is exhibited by human or animal subjects is an interesting open question.

Another way of measuring machine generalization is to test its performance under *gain regimes* outside its testing data. Since no discrepancy is enforced between the modalities, biases should be zero. Performance should be approximately optimal in the training regime, where gains ranged from 12 to 18 spikes. And indeed, Fig. 5B shows that neither the error covariance (the relative shapes of the green and black ellipses) nor the bias (the relative positions of the green and black ellipses) are noticeably worse than in the training regime until the gain ratios (PROP/VIS) reach the extreme values on the plot.

Finally, we examine machine performance under *both* input discrepancy and gain modulation, with a constant input discrepancy of 2.5 standard deviations and various gain ratios Fig. 5C. The black and green dotted lines, nearly identical, track the movement of the error means of the optimal and RBM-based estimators, respectively. This reproduces the familiar psychophysical finding that varying the relative reliability of two discrepant inputs will bias downstream activity (sc., behavior) toward the more reliable modality's estimate [1].

### Different training data

#### Learning non-flat priors

So far we trained on stimuli that were chosen uniformly in joint space, so that the posterior mean is simply the peak of the likelihood given the inputs, 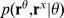. In general, of course, these quantities are distinct. Since the learning algorithm we employ is a density estimation algorithm, it is expected to reproduce the marginal density 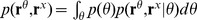, and thus should learn the prior over the stimulus as well as the likelihood. Therefore, the distribution of hidden-layer activities in the trained model will reflect both of these “input distributions,” and we should be able to decode the maximum a posteriori (MAP) estimate from the RBM. Importantly, we use the same decoding scheme employed as throughout (see **Methods**), ensuring that the prior is instantiated in the RBM rather than the decoder.

For simplicity, we chose the prior 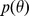 to be a tight Gaussian—with covariance on the order of the input covariances—centered in the middle of joint space (see **The optimal posterior distribution over the stimulus** in **Methods**). Thus, for a fixed stimulus, the (conditional) optimal estimator will be biased toward the center of joint space relative to that stimulus. Averaged over all stimuli, the (marginal) optimal estimator will be centrally located, but have smaller error covariance than its flat-prior counterpart—intuitively, the prior information increases the precision of the estimator.

**Figure 6:**
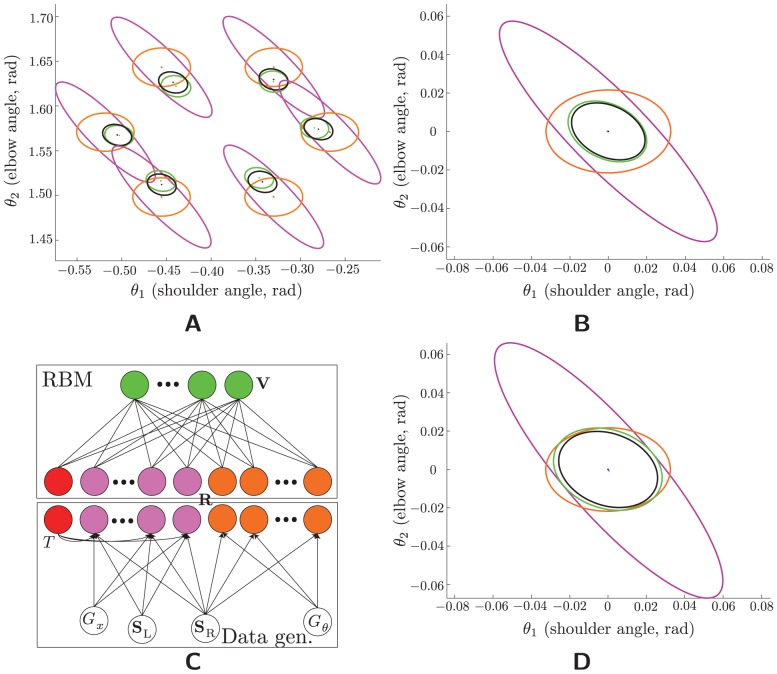
Other data distributions. (A,B) : Learning a prior. The network was trained on population codes of an underlying stimulus that was drawn from a Gaussian (rather than uniform, as in the previous figures) prior. This makes the MAP estimate tighter (cf. the black ellipses here and in Fig. 2B) —and indeed the RBM-based estimate's error covariance is correspondingly tighter. (A) Conditional estimate statistics (color scheme as throughout): The output estimates (green) have smaller covariances, but they, like the optimal estimates (black) are also biased toward the mean of the prior, located at the center of the workspace. The match between them is evidently very good. Note that the stimulus location for each of these conditional statistics is eight standard deviations from the mean of the prior—so the model has generalized well to points that constituted a trivial fraction of the training data. (B) Marginal error statistics. (C,D) : Learning that the inputs need not report the same stimulus. (C) A graphical model showing the independence relationships holding among the variables of this model. The (observed) toggle 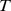 determines whether the visual population is reporting the left (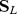) or right (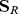) hand. (D) Marginal error statistics (colors as throughout) for the mean of the posterior distribution over the right hand. Since the visual population provides information about the right hand only 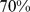 of the time, the optimal error covariance is broader than its counterpart in Fig. 2B. The RBM-based estimate again nearly matches it.

This is precisely what we see for the RBM-based estimate in Fig. 6A,B. Its conditional statistics are shown for six different fixed stimuli in Fig. 6A, along with those of the two unisensory MAP estimates and the optimal estimate (the MAP estimate given both input populations). The corresponding marginal error statistics, averaged over all stimuli under their prior distribution, are shown in Fig. 6B The RBM-based error covariance, like its optimal counterpart, is tighter than that achieved with a flat prior (cf. Fig. 2B).

#### Sometimes-decoupled inputs

We have been supposing the model to correspond to a multisensory area that combines proprioception of the (say) right hand with vision. When not looking at the right hand, then, the populations ought to be independent; and an appropriate model should be able to learn this even more complicated dataset, in which the two populations have a common source on only some subset of the total set of examples. This is another well known problem in psychophysics and computational neuroscience (see e.g. [21]). When the integrating area receives no explicit signal as to whether or not the populations are coupled, the posterior distribution over the right hand is a mixture of Gaussians, which therefore requires the encoding of numerous parameters—two means, two covariance matrices, and a mixing proportion—and is therefore rather complicated to decode. Simulations, omitted here, show that the RBM does indeed learn to encode at least the peaks of the two Gaussians.

A slightly simpler model includes among the input data an explicit signal as to whether the input populations are coupled, in our case by dedicating one neuron to reporting it. This model is shown in Fig. 6C: populations were coupled in only 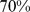 of trials; in the others, the vis (magenta) population reports the “left hand,” and the unit labelled 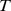 indicates this by firing at its maximum mean spike count (otherwise it is off). Derivation of the optimal error covariance for the MAP estimate is given in **Text S1**; intuitively, the model must learn to encode different distributions in its hidden units depending on whether or not 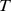 is on. When 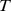 is off, these units should integrate the stimulus estimates encoded by the two populations and encode this integrated estimate (and its variance). When 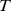 is on, it should encode the proprioceptive stimulus and the visual stimulus separately. The optimal error variance is calculated by a weighted average of the error variances in the two conditions, smaller and larger respectively, the weights being the percentage of the time each conditions occurs (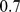 and 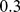, resp.). (The optimal error mean is still zero.) Fig. 6D shows that a network trained on these data—with the same architecture as throughout—again achieves this optimum.

### Other architectures

#### Hierarchical networks

**Figure 7:**
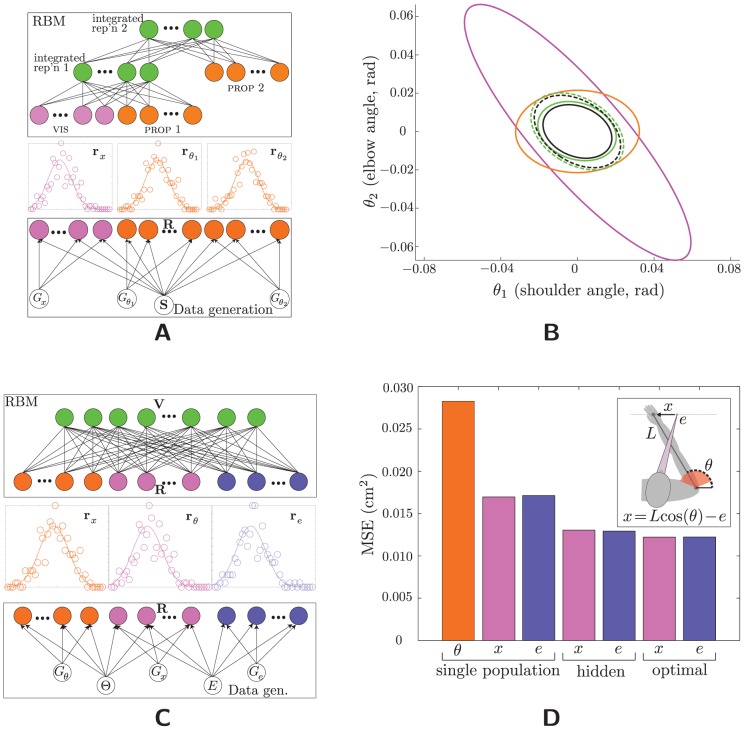
Other architectures. (A,B) A “hierarchical” network, in which a third modality must be integrated with the integrated estimate from the first stage—which is just the original model. (A) Data generation (bottom), population coding (middle), and network architecture (cf. Fig. 1A). Input units are Poisson and hidden units (green) are Bernoulli. The population codes, depicted in one dimension for simplicity, are actually 2D. Each hidden layer has half (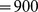) the number of units in its input layer (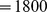). (B) Marginal error statistics. The error ellipses for Prop 1 (orange), for VIS (magenta), for both Prop 1 and VIS (dashed black), and for “integrated representation 1” (dashed green) replicate the results from Fig. 2B. Prop 2 is encoded in the same way as Prop 1 (though their activities on a given trial are never equal because of the Poisson noise), and so has identical error statistics (orange). Conditioning on this third population in addition to the other two shrinks the optimal error covariance (solid black), and the estimate decoded from “integrated representation 2” (solid green) is correspondingly smaller as well, and again nearly optimal. (C,D) Coordinate transformation. (C) Data generation (bottom), population coding (middle), and network architecture (top). Each input population (bottom panel, color coded) depends on its own gain; whereas, both Prop (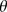, orange) and vis (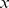, magenta) depend on the stimulus (hand position), and both vis and eye (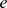, blue) depend on gaze angle. (D) Mean square errors. The RBM-based estimates have nearly minimal MSEs, demonstrating that these estimates are nearly equal to the mean of the true posterior distribution. Inset: the physical setup corresponding to coordinate transformation. Red shading denotes joint limits; the black line denotes the origin of joint space.

A plausible neural model of multisensory integration will be *composable* in the sense that the integrating neurons can themselves serve as an input population for further integration with, e.g., another modality. Fig. 7A illustrates the architecture of one such network. As above, input layers are Poisson, hidden layers are Bernoulli. The first RBM is the same as in the foregoing results; the second was trained on an input layer comprising the hidden-layer population of the first RBM (“integrated representation 1”) and a new input population (“PROP 2”), which for simplicity encodes joint angles, just as the first-layer proprioceptive population (“PROP 1”) does—though of course the population activities are different, since these are noisy. The second population also has a different gain on each trial (see the bottom panel of Fig. 7A).

Again we focus on the error statistics of the posterior mean (Fig. 7B). Both integrated representation 1 (using two inputs) and integrated representation 2 (using all three inputs) approach their optimal values. Although these error statistics are direct measures of posterior-mean encoding only, that the posterior variance is being encoded is demonstrated indirectly, as well: Proper integration at the second level requires variance information to be encoded in the first hidden layer. The (nearly) optimal error statistics for the second layer show that indeed the posterior variance information is encoded on a per-trial basis in the (first) hidden layer.

#### Coordinate transformation

We consider now another, seemingly different, computational problem studied in the sensorimotor literature, coordinate transformation (sometimes called “sensory combination” [22]). In general, the relationship between proprioception and visually-encoded stimuli is mediated by other quantities—gaze angle, head-to-body orientation, body-to-arm orientation, etc. —which are themselves random variables. In the simplest version, the relationship of vision to proprioception depends only upon gaze position, 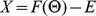, and the “stimuli” consist of two independent random variables 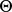 and *E* [23]. Fig. 7C depicts a probabilistic graphical model for this scenario, along with the RBM that is to learn these data (cf. Fig. 1A). The optimality equations are slightly more complicated for this problem (see **Coordinate Transformations** in **Text S1**), but conceptually similar to that of simple multisensory integration (Eq. 3).

In this model, the proprioceptive population is responsible for a larger space than either of the other two variables, a consequence of our choice to sample in the space of the latter (see Fig. S2A and related discussion in **Tuning of the coordinate-transforming neurons** in **Text S1**). Allocating to each population the same number of neurons, while also demanding that the 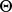 variance be small enough for its contribution to affect appreciably the integrated estimate, requires that we increase its relative gain; hence we let 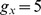, 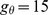, 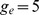. In keeping with the simple relationship just given, all variables are one-dimensional; the network allocates 60 units to each, yielding 180 total input units. The hidden layer has only 160, respecting our requirement that it be smaller than the input layer. (The ratio of hidden/input was chosen with the following rationale: The input layer encodes six random variables—the three “stimuli,” *X*, 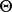, and *E*, plus their three associated gains—whereas the hidden layer needs to encode five, one of the stimuli being redundant with the other two. And indeed, using fewer than 160 hidden units yields suboptimal results. Cf. the “standard” network, for which the input encodes six variables—the two gains and the two 2D stimuli—, and the hidden layer encodes four—two gains and a single 2D stimulus. A longer discussion of these approximate calculations can be found in **Text S1**.) Fig. 7D shows that mean square errors (MSEs) of the RBM-based estimate of the stimulus are, once again, nearly optimal given the three inputs. (We can show mean and variance together as MSE without loss of generality because the posterior mean is the unique minimizer of the MSE, so showing that the RBM-based estimator achieves minimum MSE shows that it is the posterior mean.) This demonstrates the generality of our approach, as the same network and algorithm will learn to perform multisensory integration or coordinate transformations, depending simply on its inputs (cf. the networks of [24]–[27], which are built specifically to perform coordinate transformations). Nor is there reason to believe that learnable transformations are limited to simple combinations of the form 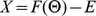, which was chosen here merely to simplify our own computations of the optimality conditions (see **Coordinate Transformations** in **Text S1**).

### Properties of the hidden units and their biological implications

We now examine some of the properties of the hidden units, especially those that electrophysiologists have focused on in multisensory neurons in rhesus macaques.

#### Integrating neurons

**Figure 8:**
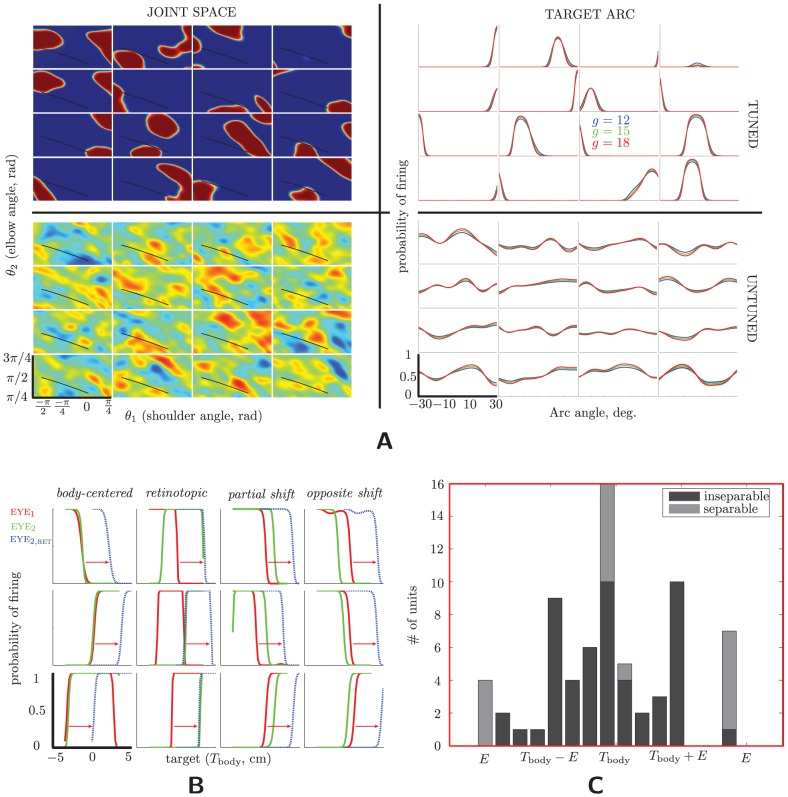
Tuning curves in the hidden layer. (A) Tuning curves for the multisensory-integration model/data (Figs. 1 and 2). The left column shows tuning curves in the space of joint angles for sixteen randomly chosen hidden units; the right column shows those same units for the arc of reach endpoints from [29]. The top row shows tuning curves for the trained model; the second row shows the same curves for the untrained model. The location of the arc in joint space is shown by the black slash through the tuning curves in the left column. Whereas the left-column tuning curves were collected for a single gain (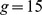), the right-column curves were collected for 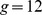 (blue), 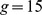 (green), and 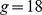 (red) (the same gain was used for both populations, vis and prop). (B) Example hidden-unit tuning curves from the coordinate transformation model for body-centered hand position (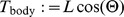; see text for details), for two different gaze positions (red and green curves). The dashed blue curves show where the red tuning curves would lie for the second gaze position if they shifted completely with the eyes, as illustrated by the red arrows, i.e. if they were retinotopic. Some cells (second column) are; some are body-centered (first column); some partially shift (third column); and some even shift in the opposite direction of the gaze angle. (C) Coordinate-transforming cells can be tuned for any of the variables on the continuum from gaze angle (*E*), to retinotopic hand position (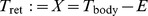), to body-centered hand position (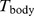), to body-centered hand position plus gaze angle (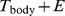). The histogram shows the distributions of such tunings in the hidden layer, using the analysis of [31].

Fig. 8A shows tuning curves for a random subset of 16 tuned hidden units in our “standard” multisensory-integration network (**Multisensory integration in the RBM**). (By “tuned” we mean neurons whose mean firing rate—i.e., probability of firing—varied by more than 0.1 over the stimulus range.) To render tuning more clearly, curves were computed noiselessly—using means in both the input and hidden layers—and with a fixed gain of 15 for both populations.

Interestingly, the two-dimensional tuning for joint angles (left column) is multimodal for many cells—although also highly structured, as apparent from comparison of tuning for the trained (upper row) and untrained (lower row) networks. Although multimodal tuning has been found in multisensory areas, for example, area VIP (see Fig. 3 of [28]), a comparison of these plots with empirical data is complicated by the fact that neurophysiologists typically do not collect tuning data over a complete planar workspace.

We therefore restrict attention to the 1D submanifold of joint space indicated by the black slash through the 2D tuning curves, corresponding to an arc in the visual space, since tuning over this range was reported in [29] (see especially the supplement) for multisensory neurons in Area 5 and MIP; we show the corresponding model tuning in the right column for the same sixteen neurons as the left column. The determination of whether or not model neurons are tuned was made along this arc (rather than the entire planar workspace); in this limited range, 137 of the 900 hidden units were tuned. Results are qualitatively similar between data and model: Along the arc, units in the trained network are unimodal and occasionally monotonic (unlike in the untrained model, bottom right). Furthermore, although none of these 16 cells exhibited bimodal tuning for this arc, from the distribution of planar tuning we expect that some cells would; and indeed a subset of cells in [29] exhibit bimodal tuning (see Fig. 5 and Supplemental Fig. 6C in the cited work).

Fig. 8A also shows how the tuning along the 1D arc depends on the input gains. Although broadly similar across gains, increasing gain does result in a subtle sharpening of the tuning curves. This can be quantified more directly by simply counting the number of active neurons for a given stimulus under different gains: sharper tuning curves will result in fewer neurons firing (though possibly more total spikes). And indeed, after sampling 15 spikes from the hidden layer, the percent of neurons firing is 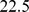, 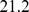, and 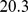, for 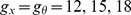, respectively. This is in contrast to the input layer, where increase in gain *increases* the number of spiking units. Sharpening is also in contrast to the theoretical predictions of [2], where the hidden layer is a probabilistic population code of the same form as the inputs, with both having the property that higher gains imply a greater number of active neurons. This feature has not been investigated directly in multisensory areas of the cortex, and presents a useful test for the model. Although the absence of sharpening would not rule out a broader class of density estimation models, it would indeed rule out this particular implementation.

#### Coordinate-transforming neurons

Investigation of multisensory tuning properties has a longer history for coordinate transformations. Here, especially in Area 5d, MIP, and VIP, neurons have been reported to encode objects in references frames intermediate between eye and body (“partially shifting receptive fields”) —i.e., the receptive field moves with the eye, but not completely; and eye position modulates the amplitude of the tuning curve (“gain fields”) [28]–[31]. As in those studies, we find examples of retinotopic, body-centered, and partially shifting receptive fields—even fields that shift opposite to the change in gaze-angle. Fig. 8B shows examples of all four types (see legend). (We conflate head-centered and body-centered tuning in what follows, since we assume a head-fixed model.).

More recently, Andersen and colleagues [31], [32] have proposed to analyze these qualitative descriptions in terms of (1) the “separability” of the tuning curve—whether it can be written 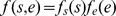; and (2) the reference frame in which the neuron is tuned—body, eye, or something intermediate—as measured by the gradient of the tuning in the 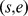 space, since the direction of steepest change indicates the strongest tuning. All and only the neurons with pure gain fields (no shift) will be separable. The extent of receptive-field shift for inseparable neurons is indicated by the gradient analysis.

We reproduce that analysis on our model here. In [31], there is a third variable in addition to hand location and gaze position, namely target. However, direct comparison between model and data can be made simply by identifying the hand and target. Finally, since all tuning curves were measured in visual space, we do the same; thus we define: 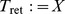, the retinotopic hand/target location in visual space; and 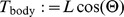, the body-centered hand/target in visual space; giving the familiar equation 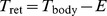. Fig. 8C shows the resulting histogram of gradient directions, which is qualitatively quite similar to its counterpart, the top panel of Figure 4 of [31]: a peak at 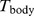, minor peaks at the other “unmixed” stimuli, with representative neurons at all stimuli combinations—except those intermediate between 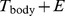 and 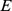, where there is a gap in the histogram.

Nevertheless, we emphasize that correspondence between model and data in Fig. 8C should be interpreted with extreme caution: it is possible to obtain different distributions of receptive-field properties with our model; see **Text S1** : **Tuning of the coordinate-transforming neurons** for further discussion.

## Discussion

We have demonstrated a neural-network model of multisensory integration that achieves a number of desirable objectives that have not been captured before in a single model: learning *de novo* to integrate the mean *and covariance* of representations of nonlinearly related inputs; learning prior distributions over the encoded stimuli; staged, hierarchical integration; and “coordinate transformations.”

Our approach is based on two central ideas. The first, following [2], is that the goal of multisensory integration is not (merely) to encode in the multisensory neurons (**v**) an optimal point estimate of the stimulus (**s**) given the activities of the input populations (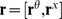); but to encode an entire (optimal) distribution, so that 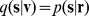. This criterion is equivalent to demanding that all the information in the input populations about the stimulus—the mean, variance, and higher cumulants, if applicable—be transferred to the multisensory neurons **v**. Behavior itself corresponds to a single point from this distribution, but the higher cumulants will be necessary for intervening computations: for example, the variance of the integrated estimate determines how to integrate it optimally with other estimates downstream (see Fig. 7).

The second central idea is that this information-retention criterion will be satisfied by the hidden or “latent” variables, **V**, of a generative model that has learned how to produce samples from the distribution of its input data, **R**, a process called latent-variable density estimation. The intuition connecting this learning problem with the seemingly very different task of multisensory integration is that being able to reproduce the input data (up to the noise) requires encoding their “hidden causes”—the features, like hand location, that vary across trials, and thus should be transmitted downstream—in the latent-variable activities. The density estimator will likewise learn to represent the statistical features that do not vary across trials, like prior information, in its weights. Since a network that has learned to reproduce its inputs efficiently will have implicitly learned the underlying relationship between their hidden causes, density estimation also naturally solves other computational problems that arise in multisensory processing: the need to perform coordinate transformations (Fig. 7C), for example, arises because a signal is available that correlates with a transformed version of other variables—like retinotopic object location with the combination of body-centered object location and gaze angle. Efficiently encoding the distribution of the larger set of variables requires learning the coordinate transformation.

With the network implementation of latent-variable density estimation, we have demonstrated how all three of these learning problems—optimal integration, the integration of prior information, and coordinate transformations—can be solved by multisensory neural circuits. We have previously argued that these three operations are exactly those required for planning multisensory-guided reaching movements [23]. There is considerable evidence for multimodal, reaching-related signals across several brain areas in the posterior parietal cortex, including Area 5d, MIP, VIP, V6, and Area 7 [33]–[38]. We propose that density estimation, driven by latent-variable learning, is the principle underlying computation performed by these areas. The fact that our network can be hierarchically composed is central to this hypothesis: these brain areas receive overlapping but distinct sets of inputs and with a rough hierarchical organization within them [39]–[43]. Density estimation on these inputs, then, is expected to yield activity patterns that are also highly overlapping but distinct, as observed, for example, in [29], [44]. We have previously argued that having a collection of such representations allows for the flexible and (nearly) optimal use of a wide range of sensory inputs [45].

### Implications of the model

One example of a statistical feature that is constant across trials is the prior distribution of the stimulus, which the network therefore learns to encode in its weights. Whether prior distributions in the brain are encoded in synaptic weights [46], [47], as a separate neural population [2], or something else again, remains an area of active research (see also **Text S1**).

An interesting consequence of the present formulation is that it renders the gains random variables (see e.g. Fig. 1A), no less than the stimulus location; that is, they represent information that is not constant across trials. This has testable implications for multisensory populations. For an 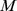-dimensional stimulus, the posterior precision (inverse covariance) of the multisensory neurons is an 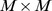 symmetric matrix and therefore has 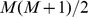 independent entries. But if the precisions of the two input populations are each functions only of a single parameter (their respective gains, reflecting the confidence in each modality), then the multisensory activities need only encode two, rather than 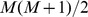, numbers on each trial. Conversely, in the case of a one-dimensional stimulus, a population of multisensory neurons ostensibly need only encode the single value of the posterior variance, 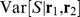, but the density-estimation approach predicts that the hidden-unit activities on a given trial will nevertheless encode both of that trial's input-population gains—and indeed they do in our model, albeit imperfectly (Fig. 3A). Testing these predictions experimentally would be straightforward—try to decode unisensory covariances from a multisensory population—but it has never been done.

The question of whether cortical circuits learn to encode any posterior covariance information at all, as opposed to merely the point estimate that psychophysical experiments elicit, is itself a crucial, open one. Of course, in theory one can always compute a posterior over the stimulus given some population activities [48]; but whether the posterior conditioned on activities deep in the hierarchy matches that conditioned on the activity in early sensory cortices, as in our model, is unknown. Our model also predicts that such constancy would emerge during learning—which could be tested, for instance, by training an animal on a novel multisensory pairing (e.g., audition and touch).

That fewer units are used to represent the same information (half as many in our simple integration model; see **Multisensory integration in the RBM**), and that the maximum spike count of each hidden neuron is bounded by the maximum mean spike count of the inputs, constrains the amount of information that can be transmitted. This forces the hidden units to represent the information more efficiently—i.e., to “integrate” it. In fact, without that constraint, no learning would be required to satisfy the information-retention criterion: A random 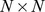 weight matrix has rank 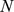 almost surely, and the neuron nonlinearities are likewise invertible, so any random set of synaptic connections would suffice (since any invertible transformation is information-preserving). We chose to constrain the multisensory representational capacity, so that the synaptic connections form an 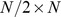 matrix, which will not in general preserve stimulus information. One promising theoretical strategy would be to take “passing on all the information” as a given, and then to seek the set of constraints—fewest spikes [49], topography [50], fewest neurons, least processing time, computational efficiency [51], etc. —that yields the most biologically realistic activity patterns in the multisensory units.

### Relationship to other work

Multisensory integration was first considered from the standpoint of information theory and unsupervised learning in [52], and in a related work [50], and our approach is similar in spirit, but with important differences. Crucially, a different objective function was minimized: integration was achieved by maximizing mutual information between the hidden/output units of two neural networks, each representing a modality, forcing these units to represent common information, the latter additionally constraining topography. In our model, contrariwise, integration is enforced indirectly, by requiring a reduced number of (hidden) units to represent the information in two populations. This allows for greater generality since it does not require foreknowledge of which populations should be forced to share information: if the information in the input populations is redundant, it will be “integrated” in the hidden units, and conversely. More recently, the idea of treating multisensory integration as a density estimation problem has been proposed independently by [53], a complementary report that explores both cognitive and neural implications of this view, without proposing an explicit neural implementation. As in [50], [52], then, no attempt is made to employ biological learning rules. Most significantly, none of these models invokes the criterion for optimal integration that we have argued to be central—the correct posterior distribution over the stimulus given hidden-unit activities (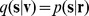, in the notation of this paper). This approach renders the combination of three signals of two independent causes—coordinate transformation—a matter simply of allowing another population to feed the hidden units; whereas the other models would require something more sophisticated.

More recent models of multisensory integration or cross-modal transformation neglect some combination of the desiderata listed in the introduction. Basis-function networks with attractor dynamics [27], [30], [54] ignore prior distributions but more significantly require hand-wiring (no learning). The models of [46] and [47] extend these attractor networks to include the learning of priors, but even these must be hand wired and so are practical only for simple representations. Other models of learning [24]–[26], [55] disregard variance information, so that what is learned is essentially a mapping of means; nor, correspondingly, do they account for the learning of priors. The probabilistic population coding model [2] makes explicit the notion of encoding a posterior, but includes no model of learning.

Finally, many authors have either anticipated [51], [56], [57] or explicitly proposed [58]–[60] that learning to process early sensory information might be viewed as forms of density estimation. Our work shows that the range of computations that can be assimilated to this statistical problem extends to the acquisition of two key operations for motor planning and control: multisensory integration, even when the underlying stimulus is distributed non-uniformly, and coordinate transformations; and further that these computations can be combined hierarchically, as is observed in the the neural circuits underlying these operations.

## Methods

Notation is standard: capital letters for random variables, lowercase for their realizations; boldfaced font for vectors, italic for scalars.

### Input-data generation

Throughout, we work with the example case of integrating two-dimensional (2D) proprioceptive and visual signals of hand location, but the model maps straightforwardly onto any pair of co-varying sensory signals. These two signals report elbow and shoulder joint angles (prop, 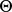), and fingertip position in Cartesian space (vis, **X**), respectively. Choosing the forward kinematics, 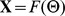, to be invertible renders the variables isomorphic, so that we can refer generically to them as a “stimulus” (**S**), independent of space. The kinematics model for most of the results has joint ranges of 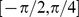 (shoulder) and 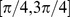 (elbow) and limb lengths of 12 (upper arm) and 20 (forearm) cm; see inset of Fig. 1A. The exception is Fig. 7C,D, in which a one-degree-of-freedom (1D) arm was used for simplicity: 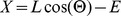, with link length 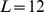 cm and joint range 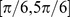, and 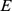 the position of the eye (eye, gaze angle). Below, we describe data generation from the 2D kinematics; the modifications for 1D are straightforward.

Each training vector consists of a set spike counts, 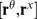, generated by choosing a random stimulus (**s**, i.e. 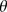 and **x**) and a random global gain for each modality (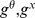), and encoding them in a populations of neurons with Gaussian tuning curves (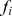) and independent Poisson spike counts—a “probabilistic population code” [2]:
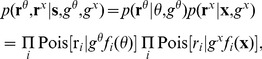
(2)as illustrated in Fig. 1A. Each gain, 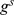, can be thought of as the confidence in its respective modality, since the posterior covariance of a single, sufficiently large population, 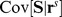, is inversely proportional to its gain [2]. The tuning curves 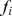 of each population are two-dimensional, isotropic, unnormalized Gaussians, whose width (variance) is 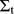, and whose centers form a regular grid over their respective spaces.

To avoid clipping effects at the edges, the space spanned by this grid of 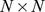 neurons is larger than the joint space (or, for VIS, than the reachable workspace). Thus the grid consists of a central “response area” whose neurons can be maximally stimulated, and a “margin” surrounding it whose neurons cannot. The margin width is four tuning-curve standard deviations (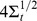), making spiking of putative neurons outside the grid extremely unlikely even for stimuli at the edge of the response area. In accordance with the broad tuning curves found in higher sensory areas and with previous models of population coding in multisensory areas [2], [27], tuning-curve widths were themselves chosen so that their full width at half maximum embraced one-sixth of the response area.

The prior over the stimulus is either uniform or Gaussian in the space of joint angles. (Implementation of the Gaussian prior is detailed in **Learning non-flat priors**.) Since both dimensions of prop space are allotted the same number of neurons (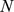) and the tuning curves are isotropic and evenly spaced, but the physical ranges of these dimensions differ (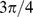 and 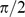 for the shoulder and elbow, resp.), the induced covariance 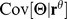 in the population code is anisotropic, being more precise in elbow than shoulder angle. The nonlinearity of the forward kinematics likewise ensures anisotropy of 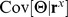; see Fig. 1A. This makes the problem more interesting, anisotropic covariances entailing, for example, optimal estimates that are not on the straight-line path between cue means (see e.g. Fig. 1 of [1]).

The priors over the gains, 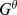 and 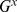, which set the maximum mean spike counts, are independent and uniform between 12 and 18 spikes. Unless otherwise noted, gains in the testing data were drawn from the same distribution as the training-data gains.

### The optimal posterior distribution over the stimulus

To show that the model works, we must compare two posterior distributions over the stimulus: the posterior conditioned on the input data, 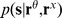
—i.e. the “true” or “optimal” posterior—and the posterior conditioned on the downstream/integrating units, 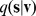 (see **The RBM**, below). That comparison is easiest to make, and to exhibit, when the optimal posterior is as simple as possible—ideally, a Gaussian, which has only two nonzero cumulants, mean and covariance. With a flat or Gaussian prior over the stimulus, the probabilistic population code that we are using does indeed have an approximately normal posterior for a unimodal population [2]; but to guarantee this for *two* populations that are encoding the stimulus in different (i.e., nonlinearly related) spaces, the unimodal posterior covariances (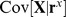 and 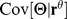) also must be small enough that typical errors lie within the linear regime of the arm kinematics (see **Text S1**). Given the gain (*G*) regime and the tuning-curve widths (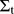), choosing 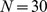 neurons in the 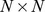 grid yields variances between 2 and 9 mm^2^ for the two populations, satisfying the requirement. These values are also comparable to empirical values for visual and proprioceptive localization variances from human psychophysics, 5 mm^2^ and 50 mm^2^, resp. [1]. These latter are in fact an upper bound, since they are with respect to behavior, the furthest downstream assay of certainty. In any case, we stress that this and other compromises of the population code with biological realism (uniform tiling of the stimulus space, identical tuning curves, etc.) serve to simplify the analyses and interpretation rather than reflecting any limitation of the neural-network model.

Now, whereas a Gaussian posterior requires a flat or Gaussian prior, such a prior in prop space will induce an irregular prior in vis space (and vice versa; see again Fig. 1A) —so there can be a Gaussian posterior only in one space. Results are therefore computed in the space of the flat or Gaussian prior. Observing these constraints, the posterior cumulants can be written:
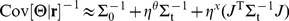
(3a)

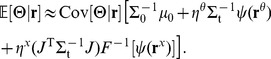
(3b)


(See **Text S1** for a derivation.) Intuitively, the posterior precision (inverse covariance, Eq. 3) is a sum of three precisions: the prior precision, 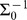; the weighted prop (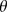) tuning-curve precision, 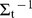; and the weighted vis (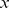) tuning-curve precision, 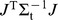. (Since the posterior is expressed over 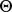 rather than **X**, the latter's precision must be warped into 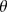-space by the Jacobian, 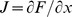, of the forward kinematics, which is evaluated at the center of mass of the proprioceptive population.) The weights are the total spike counts for each population, 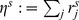, 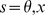. The posterior mean (Eq. 3b) is a normalized, weighted sum of three estimates: the prior mean, 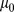; the center of mass of the 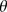 population, 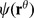; and the (transformed) center of mass of the 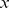 population, 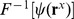. The weights are the three precisions. The center of mass 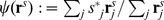, with 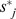 the 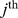 preferred stimulus, is likewise intuitive, being the maximum-likelihood estimate of the stimulus for a single population [61].

The nonlinearity (cosine) in the 1D “coordinate-transformation model” (Fig. 7C,D), 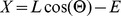, likewise allows the posterior to be normal in only one space. Since two of the variables live in Cartesian space—*X* (vis) and *E* (eye) —and only 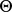 (prop) lives in joint-angle coordinates, we chose uniform priors over the former, sampling them between 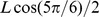 and 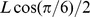, so that their sum never exceeded the bounds of the joint range (see above, **Input-data generation**). Zero in this space corresponds to hand position at the center of fixation for 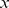, and to central fixation for *E*.

The addition of a non-flat prior (Fig. 6) will only have an appreciable effect on the posterior if the width of the prior distribution is comparable to that of the likelihoods, i.e. the single-modality localization covariances. The covariance of the prior was therefore constructed so that, along both dimensions, the extreme angles were 150 standard deviations apart—a reasonable prior distribution, perhaps, after extensive training on a reaching task to a single target location [47]. Using more realistic, broader priors would require relaxing the constraint that the optimal posterior distribution over the stimulus be Gaussian—which again we insist upon only for ease of analysis.

### The RBM

The neural circuit for sensory integration was modeled as a restricted Boltzmann machine, a two-layer, undirected, generative model with no intralayer connections and full interlayer connections (Fig. 1A, bottom right) [17], [62]. The input layer (**R**) consists of Poisson random variables, whose observed values are the population codes just described. The hidden-layer units (**V**) are binary, indicating whether or not a unit spiked on a given trial, making them Bernoulli random variables. Unless otherwise noted in the results, the number of hidden units in the model is equal to half the number of input units, i.e. the number of units in a *single* input population—thus forcing the model to represent the same information in half the number of neurons.

During RBM training [17], [62], input and hidden units reciprocally drive each other through the same weight matrix:
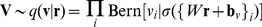
(4a)

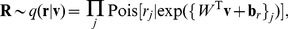
(4b)which corresponds to Gibbs sampling from the joint distribution represented by the machine. Here 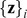 is the 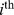 entry of the vector **z**; 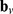 and 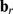 are, respectively, the vectors of biases for the hidden and observed units; 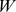 is the matrix of synaptic strengths; and 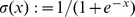 is the logistic (sigmoid) function. (The lack of intralayer connections is what allows the entire joint to be sampled in just two steps.) As in a standard stochastic neural network, each unit's mean activity is a nonlinear transformation of a weighted sum of its inputs. To ensure that this mean is in the support of its associated exponential-family distribution, the nonlinearities are chosen to be the inverse “canonical links” [63]: the logistic function for the Bernoulli hidden units, and the exponential function for the Poisson input units. (Technically, the use of Poisson input units makes the model an “exponential family harmonium” [62] rather than a restricted Boltzmann machine, which would have all Bernoulli units.) The unit's activity (presence of a spike, or spike count) is sampled from this mean.

### Training

Weights and biases were initialized randomly, after which the networks were trained on batches of 40,000 vectors, with weight changes made after computing statistics on mini-batches of 40 vectors apiece. One cycle through all 1000 mini-batches constitutes an “epoch,” and learning was repeated on a batch for 15 epochs, after which the learning rates were lowered by a factor of 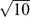. This process was repeated a total of seven times, i.e. 90 epochs, after which learning was terminated. (The number of epochs and the learning-rate annealing schedule were determined empirically.) Weight and bias changes were made according to one-step contrastive divergence [16], [17]:
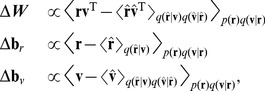
(5)where the circumflexes differentiate the zeroth (no hat) and first (hat) steps of Gibbs sampling. That is, the input data (**r**) are propagated up into the multisensory (hidden) layer (**v**), back down into the input units (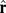), then back up into the multisensory neurons (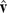); see Fig. 1B. This is repeated for all the data (that is, for each 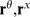 drawn from Eq. 2, for each stimulus and set of gains drawn from 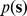 and 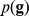). The change in the weight connecting neuron 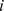 to neuron 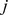 is thus proportional to the difference between the first and second pair of correlations between them—a Hebbian and an anti-Hebbian term. This rule approximates gradient descent on an objective function for density estimation (Hinton's “contrastive divergence” [17], or alternatively “probability flow” [64]). Although this specific learning rule has not been documented *in vivo*, it is constructed entirely of components that have been: change in firing rate based on (local) correlations between pre- and postsynaptic spike counts. Anti-Hebbian learning has been observed in a neural circuit [65], albeit not in mammalian cortex, and plausible cellular mechanisms for it have been described [66].

### Testing

After training, learning was turned off, and the network was tested on a fresh batch of 40,000 data vectors (Fig. 1B): stimuli were again drawn uniformly from the grid of joint angles, and the corresponding spike counts simulated by drawing from the two populations of Gaussian-tuned, Poisson neurons. For each input vector, hidden-layer activities were computed by drawing 15 sample vectors (from 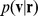) and averaging them. Since the input gains are between 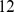 and 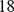, and assuming that hidden and input units integrate information over the same-sized time window from the past, this implies that hidden neurons fire no faster than input neurons—which would otherwise constitute a violation of the information bottleneck. This is essential for our task, since we require an *efficient* coding, not merely a different one.

For each trial, decoding the hidden vector consists of estimating from it the mean and covariance of the optimal posterior 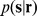
—that is, all the information in the network about the stimulus. Generally, finding a good decoder can be hard; but because the network is a generative model, we can use its generative (hidden-to-input) weights to turn the hidden vector back into expected input spike counts (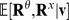)—which we know how to decode: Eq. 3. In practice, it often turns out that the weighted sum in Eq. 3b is unnecessary: the center of mass from a single (updated) population suffices. When showing results in joint angles, we take the center of mass of the prop population; likewise for Cartesian space and vis. Also, reconstruction of the total spike counts was mildly improved by first mapping them to the true (input) total spike counts via a standard neural network; in cases where this final step was applied (Fig. 3A), training and testing used different data. The posterior covariances used in Fig. 3B–D, however, did not use any such trained decoder; they were reconstructed just as the posterior means were, i.e. by using the generative weights and then applying equation Eq. 3a.

## Supporting Information

Figure S1
**Probabilistic graphical models.** The neural populations have been collapsed to single nodes. (A) A directed model for the data for *multisensory integration*. (B) A model that captures the independence statements characterizing *coordinate transformations*. (C) A model that captures the case where one population (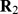) sometimes reports one stimulus, sometimes the other, as determined by 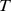.(EPS)

Figure S2
**Coordinate transformation tuning curves under different sampling schemes.** (A) The scattered black dots are sample pairs of body-centered hand position (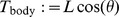) and gaze angle (*E*) that were generated from the graphical model at the bottom of Fig. 7C. Since *E* and 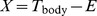 were sampled from uniform distributions on lines, the resulting space is a parallelogram. Depending on which rectangular subregion is selected (red, green, blue), different histograms of tunings result— (B), (C), and (D), respectively. See text for details of the analysis.(EPS)

Text S1 **Derivation of the optimal posterior for multisensory integration, coordinate transformation, and sometimes-decoupled inputs; notes on the fractional information loss; a rationale for the number of hidden units; and a note on the tuning of coordinate-transforming neurons.**
(PDF)
