# Supplementary material for: Learning Multisensory Integration and Coordinate Transformation via Density Estimation
Source: PLoS Comput Biol. 2013 Apr 18;9(4):e1003035. doi: 10.1371/journal.pcbi.1003035 (PMC3630212; doi:10.1371/journal.pcbi.1003035)
Supplement: Text S1 — Derivation of the optimal posterior for multisensory integration, coordinate transformation, and sometimes-decoupled inputs; notes on the fractional information loss; a rationale for the number of hidden units; and a note on the tuning of coordinate-transforming neurons. (PDF) [file pcbi.1003035.s003.pdf]

Text S1

To reiterate from the main text: the hallmark of optimality is that the "downstream" or integrating neurons encode the same information about the stimulus as their inputs. (We also demand that they do so with less capacity.) In the language of Bayesians, the posterior distribution $q(\mathbf{s}|\mathbf{v})$ over the stimulus conditioned on the activities of the integrating neurons must match the posterior $p(\mathbf{s}|\mathbf{r})$ over the stimulus conditioned on the activities of the input neurons (the "optimal posterior"). Equivalently, the cumulants of the two posteriors must match. The fewer the cumulants, then, the easier the comparison is to make, so our results are easiest to analyze if the optimal posterior is forced to be a normal distribution, which has only a mean and covariance. In that case, one need only show that these cumulants can be "recovered"—i.e., decoded—from the activities of the downstream units, $\mathbf{v}$. (Having recovered these cumulants, it is not necessary to show further that the higher-order cumulants of $q(\mathbf{s}|\mathbf{v})$ are zero: Since the integrating neurons, $\mathbf{V}$, receive all their information about $\mathbf{S}$ via the input neural activities, $\mathbf{R}$, it is not possible for them to have any additional information about the stimulus.) Below we show that our choice of encoding scheme does indeed guarantee that the optimal posterior be Gaussian: for simple multisensory integration, for the "coordinate transformation" scheme, and for a variant on a model where the two populations may report different stimuli.

Multisensory integration

We generalize the results presently, but for simplicity we start with one input population:

$$p(\mathbf{s}|\mathbf{r}, g) = \frac{1}{Z(\mathbf{r}, g)} p(\mathbf{r}|\mathbf{s}, g)p(\mathbf{s})p(g),$$

where we have assumed that stimulus intensity (encoded by gain, g) is independent of stimulus location, $\mathbf{s}$. Recall that the neurons are independent and Gaussian-tuned, and that their spike counts are Poisson-distributed. The likelihood (with $\mathbf{s}^*{}_i$ the preferred direction of neuron i, and Σ_t the tuning-curve covariance) is thus:

$$\begin{aligned}
p(\mathbf{r}|\mathbf{s}, g) &= \prod_i \mathrm{Pois}[r_i | g f_i(\mathbf{s})] \\
&= \prod_i \frac{1}{r_i!} (g f_i(\mathbf{s}))^{r_i} \exp\left\{ - g f_i(\mathbf{s}) \right\} \\
&= \left(\prod_i \frac{g^{r_i}}{r_i!} \right) \exp\left\{ - \sum_i \frac{1}{2} (\mathbf{s} - \mathbf{s}^*{}_i)^\mathrm{T} \Sigma_\mathrm{t}^{-1} (\mathbf{s} - \mathbf{s}^*{}_i) r_i \right\} \exp\left\{ - g \sum_i f_i(\mathbf{s}) \right\} \\
&\approx \left(k(g) \prod_i \frac{g^{r_i}}{r_i!} \right) \exp\left\{ - \sum_i \frac{1}{2} (\mathbf{s} - \mathbf{s}^*{}_i)^\mathrm{T} \Sigma_\mathrm{t}^{-1} (\mathbf{s} - \mathbf{s}^*{}_i) r_i \right\},
\end{aligned}$$

where we have assumed that enough uniformly tiled tuning curves exist to approximate their sum $\sum_i f_i(\mathbf{s})$ by a constant (independent of $\mathbf{s}$). (Technically, this renders all the equalities below approximate, but we refrain from using approximate equality signs going forward in order to make clear when we are making further approximations.) Now simplifying the argument $\gamma(\mathbf{s})$ of the exponential by defining the

normalized tuning covariance $\bar{\Sigma}(\mathbf{r}) := \Sigma_\mathrm{t}/\sum_i r_i$, and the center of mass $\psi(\mathbf{r}) := \sum \mathbf{s}^*{}_i r_i / \sum r_i$:

$$\begin{aligned}\gamma(\mathbf{s}) &= -\frac{1}{2}\left[\sum_i r_i \mathbf{s}^\mathrm{T}\Sigma_\mathrm{t}^{-1}\mathbf{s} - 2\mathbf{s}^\mathrm{T}\Sigma_\mathrm{t}^{-1}\sum_i \mathbf{s}^*{}_i r_i + \sum_i \mathbf{s}^{*\mathrm{T}}_i \Sigma_\mathrm{t}^{-1}\mathbf{s}^*{}_i r_i\right] \\ &= -\frac{1}{2}\left[\mathbf{s}^\mathrm{T}\bar{\Sigma}^{-1}(\mathbf{r})\mathbf{s} - 2\mathbf{s}^\mathrm{T}\bar{\Sigma}^{-1}(\mathbf{r})\psi(\mathbf{r}) + \sum_i \mathbf{s}^{*\mathrm{T}}_i \Sigma_\mathrm{t}^{-1}\mathbf{s}^*{}_i r_i\right] \\ &= -\frac{1}{2}\left[(\mathbf{s} - \psi(\mathbf{r}))^\mathrm{T}\bar{\Sigma}^{-1}(\mathbf{r})(\mathbf{s} - \psi(\mathbf{r})) + C(\mathbf{r})\right],\end{aligned}$$

where the constant C is independent of $\mathbf{s}$. Thus the single-population likelihood can be written:

$$p(\mathbf{r}|\mathbf{s}, g) = \alpha(\mathbf{r}, g)\exp\big\{-\frac{1}{2}(\mathbf{s} - \psi(\mathbf{r}))^\mathrm{T}\bar{\Sigma}^{-1}(\mathbf{r})(\mathbf{s} - \psi(\mathbf{r}))\big\}. \tag{6}$$

Assuming the prior is Gaussian with mean and covariance μ_0, Σ_0 (and normalizer Z_0):

$$\begin{aligned}p(\mathbf{s}|\mathbf{r}, g) &= \frac{\alpha(\mathbf{r}, g)p(g)}{Z(\mathbf{r}, g)Z_0}\exp\left\{-\frac{1}{2}(\mathbf{s} - \psi(\mathbf{r}))^\mathrm{T}\bar{\Sigma}^{-1}(\mathbf{r})(\mathbf{s} - \psi(\mathbf{r})) - \frac{1}{2}(\mathbf{s} - \mu_0)^\mathrm{T}\Sigma_0^{-1}(\mathbf{s} - \mu_0)\right\} \\ &= \beta(\mathbf{r}, g)\exp\left\{-\frac{1}{2}(\mathbf{s} - \hat{\mathbf{s}}_\mathrm{MAP}(\mathbf{r}))^\mathrm{T}\Sigma_\mathrm{INTEG}^{-1}(\mathbf{r})(\mathbf{s} - \hat{\mathbf{s}}_\mathrm{MAP}(\mathbf{r}))\right\},\end{aligned} \tag{7}$$

by completing the square, with $\Sigma_\mathrm{INTEG}^{-1}(\mathbf{r}) = \Sigma_0^{-1} + \bar{\Sigma}^{-1}(\mathbf{r})$ and $\hat{\mathbf{s}}_\mathrm{MAP}(\mathbf{r}) = \Sigma_\mathrm{INTEG}(\mathbf{r})(\Sigma_0^{-1}\mu_0 + \bar{\Sigma}^{-1}(\mathbf{r})\psi(\mathbf{r}))$. The normalizing constant, $\beta(\mathbf{r}, g)$, is independent of the stimulus, so the posterior must be normal. Furthermore, Eq. 7 must integrate (with respect to $\mathbf{s}$) to one, but the normalizing constant is unaffected by this integral. Therefore,

$$1 = \beta(\mathbf{r}, g)f(\mathbf{r})$$

for some function f, and so β does not actually depend on the gain, either. Thus,

$$p(\mathbf{s}|\mathbf{r}, g) = p(\mathbf{s}|\mathbf{r}) = \mathcal{N}(\hat{\mathbf{s}}_\mathrm{MAP}(\mathbf{r}), \Sigma_\mathrm{INTEG}(\mathbf{r})).$$

This justifies the naming convention $\hat{\mathbf{s}}_\mathrm{MAP}$, since the mean of a Gaussian posterior is also the MAP estimate.

If *two* populations of Gaussian-tuned, Poisson neurons ($\mathbf{r} = [\mathbf{r}_1, \mathbf{r}_2]$) encode the stimulus in the same space, the posterior:

$$\begin{aligned}p(\mathbf{s}|\mathbf{r}_1, \mathbf{r}_2) &= \frac{1}{Z(\mathbf{r}_1, \mathbf{r}_2, g_1, g_2)}p(\mathbf{r}_1, \mathbf{r}_2|\mathbf{s}, g_1, g_2)p(\mathbf{s})p(g_1)p(g_2) \\ &= \frac{1}{Z(\mathbf{r}_1, \mathbf{r}_2, g_1, g_2)}p(\mathbf{r}_1|\mathbf{s}, g_1)p(\mathbf{r}_2|\mathbf{s}, g_2)p(\mathbf{s})p(g_1)p(g_2),\end{aligned}$$

since the population activities are independent given the stimulus—and which is still Gaussian, with means and covariances adjusted in the usual way. However, if the second population encodes in a different space—if, that is, the first population uniformly tiles the stimulus in $\mathbf{s}$ and the second uniformly tiles it in $F(\mathbf{s})$ for some invertible, nonlinear function F—then the calculation is more complicated. The argument of this second population's likelihood looks like:

$$\gamma(\mathbf{s}) = -\frac{1}{2}\left[(F(\mathbf{s}) - \psi(\mathbf{r}_2))^\mathrm{T}\bar{\Sigma}^{-1}(\mathbf{r}_2)(F(\mathbf{s}) - \psi(\mathbf{r}_2))\right] + C(\mathbf{r}_2),$$

which will not, without restriction, yield a posterior Gaussian in $\mathbf{s}$. If however *the errors are small compared to the curvature of the nonlinear function*, then:

$$F(\mathbf{s}) - \psi(\mathbf{r}_2) \approx \frac{\partial F}{\partial \mathbf{s}}(\mathbf{s} - F^{-1}[\psi(\mathbf{r}_2)]). \tag{8}$$

In this linear regime, the Jacobian is approximately equal evaluated at $\mathbf{s}$ and $F^{-1}[\psi(\mathbf{r}_2)]$—a fact we make use of repeatedly. To eliminate from $\gamma(\mathbf{s})$ nonquadratic dependence on the stimulus, we exploit this approximate equality, which in (abusive) shorthand notation we write $J(\mathbf{r}_2) := \frac{\partial F}{\partial \mathbf{s}}(F^{-1}[\psi(\mathbf{r}_2)]) \approx \frac{\partial F}{\partial \mathbf{s}}(\mathbf{s}) =: J(\mathbf{s})$. The argument of the likelihood can now be rewritten as:

$$\gamma(\mathbf{s}) = -\frac{1}{2}\Big[(\mathbf{s} - F^{-1}[\psi(\mathbf{r}_2)])^{\mathrm{T}} J(\mathbf{r}_2)^{\mathrm{T}} \bar{\Sigma}^{-1}(\mathbf{r}_2) J(\mathbf{r}_2)(\mathbf{s} - F^{-1}[\psi(\mathbf{r}_2)])\Big] + C(\mathbf{r}_2).$$

And now combining this likelihood with the likelihood from the first population (smoothly tiled in $\mathbf{s}$) and the prior yields a posterior that is again Gaussian in $\mathbf{s}$, but with mean and covariance:

$$\boxed{\begin{aligned} \mathrm{Cov}[\mathbf{S}|\mathbf{r}] &\approx \Big[\Sigma_0^{-1} + \bar{\Sigma}^{-1}(\mathbf{r}_1) + J(\mathbf{r}_2)^{\mathrm{T}}\bar{\Sigma}^{-1}(\mathbf{r}_2)J(\mathbf{r}_2)\Big]^{-1} =: \Sigma_{\text{INTEG}}(\mathbf{r}) \\ \mathbb{E}[\mathbf{S}|\mathbf{r}] &\approx \Sigma_{\text{INTEG}}(\mathbf{r})\Big[\Sigma_0^{-1}\mu_0 + \bar{\Sigma}^{-1}(\mathbf{r}_1)\psi(\mathbf{r}_1) + J(\mathbf{r}_2)^{\mathrm{T}}\bar{\Sigma}^{-1}(\mathbf{r}_2)J(\mathbf{r}_2)F^{-1}[\psi(\mathbf{r}_2)]\Big] =: \hat{\mathbf{s}}_{\text{MAP}}(\mathbf{r}). \end{aligned}} \quad (9)$$

Coordinate transformations

Combining information about one variable (e.g., hand position) from two modalities (e.g., vision and proprioception) often requires information about a second variable (e.g., gaze angle). For stimuli $\mathbf{T}_i$, then, each tiled uniformly by a population of neurons, these "coordinate transformations" are of the form $h_1(\mathbf{T}_1) = h_2(\mathbf{T}_2) + h_3(\mathbf{T}_3)$, where the h_i are invertible and possibly nonlinear; or in the case of transforming retinotopic hand position into proprioceptive space via gaze angle, $X = F(\Theta) - E$. This corresponds to the graphical model in Fig. S1B (although that model is in fact more general), whose independencies we exploit below. We treat the general case in which each of i variables has been transformed by some nonlinearity h_i so that the problem can be solved once for a single variable and the other solutions given by inspection, *mutatis mutandis*. To lighten notation we write simply $\mathbf{S}_1 := h_1(\mathbf{T}_1), \mathbf{S}_2 := h_2(\mathbf{T}_2), \mathbf{S}_3 := h_3(\mathbf{T}_3)$ wherever possible, but note that the neural populations tile the stimuli uniformly in the space of the $\mathbf{T}_i$ and *not* the space of the $\mathbf{S}_i$.

Beginning again with the posterior,

$$\begin{aligned} p(\mathbf{s}_1|\mathbf{r}_1,\mathbf{r}_2,\mathbf{r}_3) &\propto p(\mathbf{r}_1,\mathbf{r}_2,\mathbf{r}_3|\mathbf{s}_1)p(\mathbf{s}_1) \\ &= p(\mathbf{r}_1|\mathbf{s}_1)\int_{\mathbf{s}_2} p(\mathbf{r}_2,\mathbf{r}_3,\mathbf{s}_2|\mathbf{s}_1)d\mathbf{s}_2 p(\mathbf{s}_1) \\ &= p(\mathbf{r}_1|\mathbf{s}_1)\int_{\mathbf{s}_2} p(\mathbf{r}_2,\mathbf{r}_3|\mathbf{s}_1,\mathbf{s}_2)p(\mathbf{s}_2|\mathbf{s}_1)d\mathbf{s}_2 p(\mathbf{s}_1) \\ &= p(\mathbf{r}_1|\mathbf{s}_1)\int_{\mathbf{s}_2} p(\mathbf{r}_2|\mathbf{s}_2)p(\mathbf{r}_3|\mathbf{s}_1,\mathbf{s}_2)p(\mathbf{s}_2)d\mathbf{s}_2 p(\mathbf{s}_1) \\ &= p(\mathbf{r}_1|\mathbf{s}_1)\int_{\mathbf{s}_2} p(\mathbf{r}_2|\mathbf{s}_2)p(\mathbf{r}_3|\mathbf{S}_3 = \mathbf{s}_1 - \mathbf{s}_2)p(\mathbf{s}_2)d\mathbf{s}_2 p(\mathbf{s}_1), \end{aligned}$$

where the last line follows because $\mathbf{R}_3$ depends on $\mathbf{S}_1$ and $\mathbf{S}_2$ only through their difference. Again employing the assumption of small errors with respect to the curvature of the nonlinearities, each likelihood is again log-quadratic in ("Gaussian in") its stimulus $\mathbf{s}_i$ (cf. Eq. 6); to wit:

$$p(\mathbf{r}_i|\mathbf{s}_i,g_i) \propto \exp\Big\{-\frac{1}{2}\Big[(\mathbf{s}_i - h_i[\psi(\mathbf{r}_i)])^{\mathrm{T}} J_i(\mathbf{r}_i)^{\mathrm{T}}\bar{\Sigma}^{-1}(\mathbf{r}_i)J_i(\mathbf{r}_i)(\mathbf{s}_i - h_i[\psi(\mathbf{r}_i)])\Big]\Big\},$$

for $i = 1, 2, 3$, and where $J_i = [\partial h_i / \partial \mathbf{t}_i]^{-1}$, evaluated at the appropriate center of mass. From here on we employ:

$$\begin{aligned} \Sigma_i^{-1}(\mathbf{r}_i) &:= J_i(\mathbf{r}_i)^{\mathrm{T}} \bar{\Sigma}^{-1}(\mathbf{r}_i) J_i(\mathbf{r}_i), \\ \phi_i(\mathbf{r}_i) &:= h_i[\psi(\mathbf{r}_i)], \end{aligned} \tag{10}$$

and suppress $\mathbf{r}$-dependency whenever possible. The second density under the integral is thus:

$$p(\mathbf{r}_3|\mathbf{S}_3 = \mathbf{s}_1 - \mathbf{s}_2, g_3) \propto \exp\left\{ -\frac{1}{2}\left[(\mathbf{s}_1 - \mathbf{s}_2 - \phi_3)^{\mathrm{T}} \Sigma_3^{-1} (\mathbf{s}_1 - \mathbf{s}_2 - \phi_3) \right] \right\}.$$

To use these densities, we need to "unmarginalize" the gains. Resuming the progression:

$$\begin{aligned} p(\mathbf{s}_1|\mathbf{r}_1, \mathbf{r}_2, \mathbf{r}_3) &\propto p(\mathbf{r}_1|\mathbf{s}_1) \int_{\mathbf{s}_2} p(\mathbf{r}_2|\mathbf{s}_2) p(\mathbf{r}_3|\mathbf{S}_3 = \mathbf{s}_1 - \mathbf{s}_2) p(\mathbf{s}_2) d\mathbf{s}_2 p(\mathbf{s}_1) \\ &= \int_{\mathbf{g}} p(\mathbf{r}_1|\mathbf{s}_1, g_1) \int_{\mathbf{s}_2} p(\mathbf{r}_2|\mathbf{s}_2, g_2) p(\mathbf{r}_3|\mathbf{S}_3 = \mathbf{s}_1 - \mathbf{s}_2, g_3) p(\mathbf{s}_2) d\mathbf{s}_2 p(\mathbf{s}_1) p(g_1) p(g_2) p(g_3) d\mathbf{g} \\ &\propto \int_{\mathbf{g}} p(\mathbf{r}_1|\mathbf{s}_1, g_1) \int_{\mathbf{s}_2} p(\mathbf{r}_2|\mathbf{s}_2, g_2) p(\mathbf{r}_3|\mathbf{S}_3 = \mathbf{s}_1 - \mathbf{s}_2, g_3) d\mathbf{s}_2 d\mathbf{g}, \end{aligned}$$

where in the last line we have now assumed flat priors over $\mathbf{S}_1$, $\mathbf{S}_2$, and the gains, in order to simplify the final result. That leaves two densities under the integral over $\mathbf{S}_2$. Combining them by again completing the square, and then integrating, yields (after some algebra),

$$p(\mathbf{r}_2, \mathbf{r}_3|\mathbf{s}_1) \propto \exp\left\{ -\frac{1}{2}\left[(\phi_2 + \phi_3 - \mathbf{s}_1)^{\mathrm{T}} (\Sigma_2 + \Sigma_3)^{-1} (\phi_2 + \phi_3 - \mathbf{s}_1) \right] \right\}.$$

Since the gains do not appear in factors that reference $\mathbf{s}_1$ (i.e., the exponential factors; cf. Eq. 7), they integrate out of the equation, leaving only the product of yet another pair of log-quadratic (in $\mathbf{s}_1$) terms: one, from the above, with covariance $(\Sigma_2 + \Sigma_3)$ and about $\phi_2 + \phi_3$; and one from $p(\mathbf{r}_1|\mathbf{s}_1, g_1)$, with covariance Σ_1 and about ϕ_1. So the posterior over $\mathbf{S}_1$ is again (approximately) Gaussian, with mean and covariance:

$$\boxed{\begin{aligned} \Sigma_{\text{INTEG},1}^{-1}(\mathbf{r}) &\approx \Sigma_1^{-1}(\mathbf{r}_1) + (\Sigma_2(\mathbf{r}_2) + \Sigma_3(\mathbf{r}_3))^{-1} \\ \hat{\mathbf{s}}_{\text{MAP},1}(\mathbf{r}) &\approx \Sigma_{\text{INTEG},1}(\mathbf{r}) \left[\Sigma_1^{-1}(\mathbf{r}_1)\phi_1(\mathbf{r}_1) + (\Sigma_2(\mathbf{r}_2) + \Sigma_3(\mathbf{r}_3))^{-1}(\phi_2(\mathbf{r}_2) + \phi_3(\mathbf{r}_3)) \right]. \end{aligned}} \tag{11}$$

Together with the definition of the transformed covariances and centers of mass in Eq. 10, these equations define the optimal posterior over one variable in a coordinate-transformation scheme. Writing the posteriors for the other two variables is essentially a matter of swapping indices. However, it must be emphasized that it will not in general be possible to have uniform priors over all three variables: even in the simplest case of $\mathbf{T}_1 = \mathbf{T}_2 + \mathbf{T}_3$, at most two of the variables can be uniformly distributed. In the simulations discussed in the main text, the transformation $X = F(\Theta) - E$ was used, and the data were chosen uniformly in X and E. This renders the prior distribution over Θ non-uniform, so optimal estimators were computed only for the former two variables.

Decoupled populations

Sometimes I'm not looking at my hand—on which trials $\mathbf{X}$ and $\boldsymbol{\Theta}$ are independent of each other. Say for concreteness that $\boldsymbol{\Theta}$ is the right-hand position in proprioceptive space, and that $\mathbf{X}$ is the visual

position of either the right or left hand, depending on which one I'm looking at. Intuitively, the posterior distribution over right-hand position in this case ought to be a mixture of two (normal) distributions: one given by the optimal combination of the two populations' stimulus estimates (as above), and one given by $\mathbf{\Theta}$ alone. The mixture weights for the two Gaussians ought to be the probability of the populations coding the same or different stimuli, respectively.

Mathematically, this can be shown as follows. Fig. S1C depicts a graphical model corresponding to this problem, where the "toggle" T determines which hand is in view. For simplicity, we take the populations to be encoding right-hand position in the same way, although a nonlinearity can be accounted for straightforwardly as above. For the same reason, we don't explicitly model the gains (as above), but they enter the equations transparently. The graph gives us the following factorization of the joint:

$$p(\mathbf{s}_1, \mathbf{s}_2, t, \mathbf{r}_1, \mathbf{r}_2) = p(\mathbf{r}_1|\mathbf{s}_1)p(\mathbf{r}_2|\mathbf{s}_1, \mathbf{s}_2, t)p(\mathbf{s}_1)p(\mathbf{s}_2)p(t); \tag{12}$$

and the toggling further entails:

$$\begin{aligned} p(\mathbf{r}_2|\mathbf{s}_1, \mathbf{s}_2, T = 1) &= \prod_i \text{Pois}[r_i|gf_i(\mathbf{s}_1)] = p(\mathbf{r}_2|\mathbf{s}_1, T = 1), \\ p(\mathbf{r}_2|\mathbf{s}_1, \mathbf{s}_2, T = 0) &= \prod_i \text{Pois}[r_i|gf_i(\mathbf{s}_2)] = p(\mathbf{r}_2|\mathbf{s}_2, T = 0). \end{aligned} \tag{13}$$

From these equations, it also follows that when $T = 0$, $\mathbf{R}_2$ is independent of $\mathbf{S}_1$:

$$\begin{aligned} p(\mathbf{r}_2|\mathbf{s}_1, T = 0) &= \int_{\mathbf{s}_2} p(\mathbf{r}_2, \mathbf{s}_2|\mathbf{s}_1, T = 0)d\mathbf{s}_2 \\ &= \int_{\mathbf{s}_2} p(\mathbf{r}_2|\mathbf{s}_1, \mathbf{s}_2, T = 0)p(\mathbf{s}_2|\mathbf{s}_1, T = 0)d\mathbf{s}_2 \\ &= \int_{\mathbf{s}_2} p(\mathbf{r}_2|\mathbf{s}_2, T = 0)p(\mathbf{s}_2|T = 0)d\mathbf{s}_2 \\ &= p(\mathbf{r}_2|T = 0). \end{aligned} \tag{14}$$

Now, if the populations were *known* (rather than inferred) to be decoupled—i.e., if $T = 0$ were observed—the posterior over the right-hand stimulus could be computed directly (using Eq. 14):

$$\begin{aligned} p(\mathbf{s}_1|\mathbf{r}_1, \mathbf{r}_2, T = 0) &\propto p(\mathbf{r}_1, \mathbf{r}_2|\mathbf{s}_1, T = 0)p(\mathbf{s}_1|T = 0) \\ &= p(\mathbf{r}_1|\mathbf{s}_1, T = 0)p(\mathbf{r}_2|\mathbf{s}_1, T = 0)p(\mathbf{s}_1) \\ &= p(\mathbf{r}_1|\mathbf{s}_1)p(\mathbf{r}_2|T = 0)p(\mathbf{s}_1) \\ &\propto p(\mathbf{r}_1|\mathbf{s}_1)p(\mathbf{s}_1) \\ &\propto \mathcal{N}(\mathbf{s}_1; \hat{\mathbf{s}}_1(\mathbf{r}_1), \Sigma_1(\mathbf{r}_1)), \end{aligned} \tag{15}$$

where $\hat{\mathbf{s}}_1(\mathbf{r}_1)$ is the single-population estimate of $\mathbf{s}_1$ (adjusted by the prior), and $\Sigma_1(\mathbf{r}_1)$ is likewise the posterior covariance using just the $\mathbf{r}_1$ population (and again prior precision). The last line follows from the penultimate one in the usual way, $p(\mathbf{r}_1|\mathbf{s}_1, T = 0)$ and $p(\mathbf{s}_1)$ being again log-quadratic in the stimulus (as in the standard case). A similar calculation yields the posterior given that the populations *are* coupled:

$$\begin{aligned} p(\mathbf{s}_1|\mathbf{r}_1, \mathbf{r}_2, T = 1) &\propto p(\mathbf{r}_1, \mathbf{r}_2|\mathbf{s}_1, T = 1)p(\mathbf{s}_1|T = 1) \\ &= p(\mathbf{r}_1|\mathbf{s}_1, T = 1)p(\mathbf{r}_2|\mathbf{s}_1, T = 1)p(\mathbf{s}_1) \\ &= p(\mathbf{r}_1|\mathbf{s}_1)p(\mathbf{r}_2|\mathbf{s}_1, T = 1)p(\mathbf{s}_1) \\ &\propto \mathcal{N}(\mathbf{s}_1; \hat{\mathbf{s}}(\mathbf{r}_1, \mathbf{r}_2), \Sigma(\mathbf{r}_1, \mathbf{r}_2)), \end{aligned} \tag{16}$$

where now $\hat{\mathbf{s}}(\mathbf{r}_1, \mathbf{r}_2)$ is the dual-population (integrated) estimate of $\mathbf{s}_1$, and $\Sigma(\mathbf{r}_1, \mathbf{r}_2)$ the covariance after combining both populations (and the prior).

Writing the full posterior is now straightforward:

$$\begin{aligned} p(\mathbf{s}_1|\mathbf{r}_1, \mathbf{r}_2) &= \sum_t p(\mathbf{s}_1|\mathbf{r}_1, \mathbf{r}_2, t)p(t|\mathbf{r}_1, \mathbf{r}_2) \\ &= p(\mathbf{s}_1|\mathbf{r}_1, \mathbf{r}_2, T = 0)p(T = 0|\mathbf{r}_1, \mathbf{r}_2) + p(\mathbf{s}_1|\mathbf{r}_1, \mathbf{r}_2, T = 1)p(T = 1|\mathbf{r}_1, \mathbf{r}_2) \\ &= p(T = 0|\mathbf{r}_1, \mathbf{r}_2)\mathcal{N}(\mathbf{s}_1; \hat{\mathbf{s}}_1(\mathbf{r}_1), \Sigma_1(\mathbf{r}_1)) + p(T = 1|\mathbf{r}_1, \mathbf{r}_2)\mathcal{N}(\mathbf{s}_1; \hat{\mathbf{s}}(\mathbf{r}_1, \mathbf{r}_2), \Sigma(\mathbf{r}_1, \mathbf{r}_2)), \end{aligned} \tag{17}$$

a Gaussian mixture model.

In general, then, the posterior distribution can be summarized by five parameters: $\hat{\mathbf{s}}_1(\mathbf{r}_1)$, $\Sigma_1(\mathbf{r}_1)$, $\hat{\mathbf{s}}(\mathbf{r}_1, \mathbf{r}_2)$, $\Sigma(\mathbf{r}_1, \mathbf{r}_2)$, and $p(T = 0|\mathbf{r}_1, \mathbf{r}_2) =: \alpha$ (since $p(T = 1|\mathbf{r}_1, \mathbf{r}_2) = 1 - \alpha$). To simplify decoding, however, we allow T to be observed, making α either 1 or 0 on that trial, and rendering the posterior on each trial a single Gaussian, and hence with just two parameters to encode.

Fractional information loss

In **Multisensory integration in the RBM**, we measured how well information had been transmitted to the hidden layer my using the fractional information loss, as a function of the gains $\mathbf{g}$:

$$\mathrm{FIL}(\mathbf{g}) := \frac{\Big\langle \mathrm{KL}\{p(\mathbf{s}|\mathbf{r})||q(\mathbf{s}|\mathbf{v})\} \Big\rangle_{q(\mathbf{v}|\mathbf{r})p(\mathbf{r}|\mathbf{g})}}{\Big\langle \mathrm{KL}\{p(\mathbf{s}|\mathbf{r})||p(\mathbf{s})\} \Big\rangle_{p(\mathbf{r}|\mathbf{g})}}. \tag{18}$$

(This measure was helpfully suggested by Jeff Beck [personal communication].) To match our testing phase, $q(\mathbf{v}|\mathbf{r})$ is interpreted to mean the distribution over $\mathbb{Z}_{15}^N$, the space of 15-ary vectors that results from sampling the hidden layer 15 times for a given $\mathbf{r}$. The angle brackets denote sample averages. The denominator can also be thought of (perhaps more intuitively) as mutual information between the stimulus and input populations, for a given gain:

$$\begin{aligned} \Big\langle \mathrm{KL}\{p(\mathbf{s}|\mathbf{r})||p(\mathbf{s})\} \Big\rangle_{p(\mathbf{r}|\mathbf{g})} &= \int_\mathbf{r} p(\mathbf{r}|\mathbf{g}) \int_\mathbf{s} p(\mathbf{s}|\mathbf{r}) \log \frac{p(\mathbf{s}|\mathbf{r})}{p(\mathbf{s})} d\mathbf{s}d\mathbf{r} \\ &= \int_\mathbf{r} \int_\mathbf{s} p(\mathbf{s}, \mathbf{r}|\mathbf{g}) \log \frac{p(\mathbf{s}, \mathbf{r}|\mathbf{g})}{p(\mathbf{s}|\mathbf{g})p(\mathbf{r}|\mathbf{g})} d\mathbf{s}d\mathbf{r} \\ &= I(\mathbf{S}, \mathbf{R}|\mathbf{g}), \end{aligned}$$

where the second line follows because $p(\mathbf{s}|\mathbf{r}, \mathbf{g}) = p(\mathbf{s}|\mathbf{r})$ in our encoding scheme. In the main text, we pointed out that this quantity ranges between 0 (in the best case, when $q(\mathbf{s}|\mathbf{v}) = p(\mathbf{s}|\mathbf{r})$) and 1 (in the worst case, when none of the information in $\mathbf{R}$ about $\mathbf{S}$ is transmitted to $\mathbf{V}$, and $q(\mathbf{s}|\mathbf{v}) = p(\mathbf{s})$). Mathematically, there are of course distributions, $q(\mathbf{s})$, for which the expression is greater than 1, but $q(\mathbf{s}|\mathbf{v})$ will never be equal to such a distribution, since even for a completely uninformative likelihood $q(\mathbf{v}|\mathbf{r})$, the posterior is never less informative than the prior, $p(\mathbf{s})$.

Rationale for the number of hidden units

The point generally of limiting the number of hidden units can be put intuitively as follows. A hidden layer which made use of as many (or more) units as the input layer to represent the same information might have "integrated" it in the sense that single hidden units respond to both (e.g.) visual and proprioceptive

inputs, but would not obviously have achieved anything thereby. More precisely, if there were as many (or more) hidden as input units, information transmission (our criterion for successful integration) would be satisfied trivially, for the reasons given in the last paragraph of "Implications of the Model" in the **Discussion**.

Other than being less than the number of inputs ($2N$, as we have referred to it throughout the main text), how should the number of hidden units be chosen? Empirical investigation in Fig. 4 showed that the error in the multisensory-integration model, Fig. 1A, becomes noticeable (although not very large) for fewer than N units. Now, the input layer encodes six variables: two gains, and two two-dimensional stimuli; whereas the hidden layer need only encode four: two gains, and *one* two-dimensional stimulus. (The number of variables the hidden units need to encode is the number of true underlying dimensions, which can be found e.g. by counting up the number of hidden variables in the "data generation" part of the schematics of Fig. 1A and Fig. 7C for multisensory integration and coordinate transformation, respectively—with the proviso that $\mathbf{S}$ must be counted twice in the former, because it is a two-dimensional variable.) That the hidden layer can be less than 4/6 the size of the input layer presumably shows that the latter has excess (unused) capacity.

In the coordinate-transformation model, Fig. 7C, the hidden layer needs to encode 5/6 as many things as the input layer does: two stimuli and three gains vs. three stimuli and three gains. With 180 input units, a conservative estimate would then allocate $(5/6)180 = 150$ units to the hidden layer. In fact, we used 160—but the numbers are in the right range.

Nevertheless, in practice, we found it difficult to train good coordinate-transforming models with fewer than 160 units. Now, it seems plausible that input layer of this model, encoding 1D variables, has less excess capacity than the multisensory-integration model, a greater number of input neurons being active on any given trial (due to a kind of reverse "curse of dimensionality"). However, we also suspect that the apparent need for more hidden units in the case of coordinate transformation may reflect greater difficulty in learning, since the computation involves learning a non-invertible map, $x = F(\theta) - e$. It seems noteworthy in this respect that many of the neural-network models in the literature for coordinate transformation in fact require $(2N)^2$ hidden units.

The relative capacities of the input and hidden layers is furthermore complicated by the fact that the former are Poisson and the latter Bernoulli. We did limit the number of samples of the Bernoulli units to the maximum mean (across the population) of the Poisson units, but this does not prevent the hidden layer from averaging a higher total spike count. The PPCs for multisensory integration of [1] likewise use half the number units in the multisensory layer, but an equal average number of spikes. In any case, the question of minimum capacity is interesting, but peripheral to our investigation in the main text.

Tuning of the coordinate-transforming neurons

In Fig. 7C of the main text (**Other architectures**), we presented a histogram of the tuning properties of the hidden units in the model of coordinate transformation using the analysis of [2]. Here we discuss several caveats.

The model of coordinate transformation, as lately discussed, has the form $\mathbf{S}_1 = \mathbf{S}_2 + \mathbf{S}_3$, or in our case, $X = L\cos(\Theta) - E$. In order to compare our results with [3], we redefined all our variables in visual space: $T_{\text{ret}} := X$ and $T_{\text{body}} := L\cos(\Theta)$, the target/hand position in the retinotopic and body-centered references frames, respectively. Recall from **Methods** that we generated stimulus data by sampling uniformly in T_{ret} (i.e, X) and E according to:

$$T_{\text{ret}} \sim \mathcal{U}\Big(-L\cos(5\pi/6)/2, L\cos(\pi/6)/2\Big) = \mathcal{U}\Big(\frac{-L\sqrt{3}}{4}, \frac{L\sqrt{3}}{4}\Big)$$

$$E \sim \mathcal{U}\Big(-L\cos(5\pi/6)/2, L\cos(\pi/6)/2\Big) = \mathcal{U}\Big(\frac{-L\sqrt{3}}{4}, \frac{L\sqrt{3}}{4}\Big),$$

in order to respect the joint limits on θ, $[\pi/6, 5\pi/6]$—which translate into $T_{\text{body}} \in [-L\sqrt{3}/2, L\sqrt{3}/2]$. That means that the gaze-position and body-centered-target pair, (E, T_{body}), is distributed (uniformly) on a parallelogram with corners at:

$$
\begin{aligned}
(e_{\text{min}}, e_{\text{min}} + x_{\text{min}}) &= \left(\frac{-L\sqrt{3}}{4}, \frac{-L\sqrt{3}}{2}\right) \\
(e_{\text{min}}, e_{\text{min}} + x_{\text{max}}) &= \left(\frac{-L\sqrt{3}}{4}, 0\right) \\
(e_{\text{max}}, e_{\text{max}} + x_{\text{min}}) &= \left(\frac{L\sqrt{3}}{4}, 0\right) \\
(e_{\text{max}}, e_{\text{max}} + x_{\text{max}}) &= \left(\frac{L\sqrt{3}}{4}, \frac{L\sqrt{3}}{2}\right);
\end{aligned}
$$

see Fig. S2A.

The analysis method of [2,3], however, requires samples from a rectilinear region in (E, T_{body}) space, since it proceeds by taking a singular-value decomposition of a matrix of firing rates on discrete points in that region. This obliges us to select, with no obvious hard rule, a rectilinear subregion from the rhombus of Fig. S2A. Three example regions are shown in that figure, and the corresponding tuning histograms in Fig. S2B-D. It is clear that the choice of subregion greatly influences the histogram. Nor is this problem obviously confined to analysis of the model; the experimental choice of gaze angles and target positions will, presumably, have the same effect. In the main text, we used the red rectangle, in order to cover the same fraction of each dimension, E and T_{body}, the maximal such fraction being 1/3; but this is certainly not the only plausible rationale.

In fact, we have additional reservations about the analysis method, as well, but for brevity we merely note here that we used, following [3], a 4×4 grid of points in (E, T_{body}) space. It seems clear that this low resolution can bias the resulting distribution of tuning; and in fact we do find (not shown) that increasing the grid to 40×40 has a visible effect on the histogram.

Last, and parenthetically, we note the designation "coordinate transformation" is far from universal: Landy calls it "promotion" [4], Ernst and colleagues [5] propose to call it a kind of "sensory combination," and Beck and coworkers call it "marginalization" [6], since the distribution over $\mathbf{s}_1$ is found by marginalizing $\mathbf{s}_2$ and $\mathbf{s}_3$ out of the joint distribution over $p(\mathbf{s}_1, \mathbf{s}_2, \mathbf{s}_3|\mathbf{r})$. Likewise, what we call "multisensory integration," Ernst et al. call "sensory integration" and Ma and Beck call "cue combination" [1].

References

1. Ma WJ, Beck JM, Latham PE, Pouget A (2006) Bayesian inference with probabilistic population codes. Nature Neuroscience 9: 1423–1438.

2. Pesaran B, Nelson MJ, Andersen RA (2006) Dorsal premotor neurons encode the relative position of the hand, eye, and goal during reach planning. Neuron 51: 125–34.

3. Bremner LR, Andersen RA (2012) Coding of the Reach Vector in Parietal Area 5d. Neuron 75: 342–351.

4. Landy MS, Maloney LT, Johnston EB, Young M (1995) Measurement and modeling of depth cue combination: in defense of weak fusion. Vision research 35: 389–412.

5. Ernst MO, Bülthoff HH (2004) Merging the senses into a robust percept. Trends in cognitive sciences 8: 162–9.

6. Beck JM, Latham PE, Pouget A (2011) Marginalization in neural circuits with divisive normalization. The Journal of neuroscience : the official journal of the Society for Neuroscience 31: 15310–9.
